# Trapped in declining occupations: Barriers to worker mobility in a changing economy

**DOI:** 10.1126/sciadv.adx3471

**Published:** 2026-03-06

**Authors:** Xi Song, Jennie E. Brand, Sukie Xiuqi Yang, Michael Lachanski

**Affiliations:** ^1^ Department of Sociology, Columbia University, New York, New York, USA.; ^2^ Department of Sociology, University of California, Los Angeles, California, USA.; ^3^ Department of Sociology, University of Pennsylvania, Philadelphia, Pennsylvania, USA.; ^4^ McDonough School of Business, Georgetown University, Washington, D.C., USA.

## Abstract

The US has undergone substantial changes in jobs, occupations, and mobility over the past two decades. Using administrative data from the US Occupational Outlook Handbook (2000 to 2020), we examine how immediate and projected occupational restructuring affects workers’ mobility. In an update to prior research, we find that workers in both growing and declining occupations experience greater mobility than those in stable occupations. However, the direction of movement varies. Workers in declining occupations often move laterally into other declining occupations, with nearly 60% experiencing downward mobility. In contrast, growing occupations offer better prospects for upward mobility, particularly for workers transitioning from declining to growing occupations, where almost 50% enter higher-paying occupations. However, these moves to emerging jobs are relatively rare, accounting for only 5% of all occupational movements. These results highlight how recent shifts in the occupational structure exacerbate existing disadvantages for workers facing declining job opportunities.

## Teaser

Recent occupational restructuring is widening the mobility opportunity gap between workers in growing and declining occupations.

## INTRODUCTION

In his classic book *When Work Disappears* (*1*), W. J. Wilson argued that problems endemic to US society, particularly in its inner cities—from poverty and family instability to substance abuse and crime—have a common cause: the disappearance of manufacturing jobs in the wake of economic globalization and evolving technologies. A large body of literature has documented the economic shocks experienced by workers due to technological change, foreign trade, offshoring and outsourcing, and consumption shifts (*2*–*8*). In recent decades, two new trends have emerged in occupational restructuring. First, the increased use of automation and artificial intelligence is encroaching on a wider range of jobs, not only in manufacturing but also more broadly in professional, technological, clerical, sales, and service occupations (*9*–*12*). Second, many new jobs are emerging, especially in high-tech, biomedical, healthcare, data science, and new energy occupations (*13*, *14*). We investigate whether recent occupational restructuring has created new patterns of inequality in occupational mobility between workers in occupations with job expansion and contraction.

To examine worker mobility, previous sociological research has distinguished between two types of mobility: structural versus exchange mobility (*15*–*17*). Structural or forced mobility reflects differences in individuals’ occupational distributions (or marginals) between two points in time or across two generations. This type of mobility happens because of shifts in job types and job availability across different periods. Over time, changes in occupational structure can lead individuals to move into or out of different kinds of jobs in response to evolving labor market conditions. Circulation, pure, relative, or exchange mobility, on the other hand, refers to the part of mobility that remains after accounting for the changes necessitated by shifts in occupational structure. This type of mobility occurs because of factors other than structural changes in the labor market. Circulation mobility is generally attributed to the decisions of employers or individuals. It represents social mobility driven by more microlevel factors as opposed to broader changes in job opportunities.

Traditional sociological studies of social mobility have predominantly focused on trends in exchange mobility, which was considered an indicator of “a society’s openness” (*18*). A commonly held view is that, in an ideal world with a fixed occupational structure, a higher level of exchange social mobility—i.e., greater openness—gives individuals more opportunities to switch occupations (*19*). However, this view overlooks how occupational restructuring itself creates unequal opportunities, making it easier for workers in some occupations to move than others. To measure occupational restructuring explicitly, Hauser *et al.* (*20*) introduced the index of dissimilarity estimated from the marginal distributions of mobility tables. Subsequent studies have extended this approach by developing additional measures, all of which still rely on marginal distributions (*17*, *21*).

Previous approaches to structural mobility have two widely recognized limitations. First, the marginal distributions in mobility tables derived from observational data can yield biased estimates of structural change. This bias arises when small occupations are excluded from surveys or when the sample size fails to capture broader shifts—especially at highly disaggregated, microclass occupational levels (*22*–*25*). To more accurately capture structural changes, we use administrative data from the Bureau of Labor Statistics (BLS), which provide detailed, job-level information within occupations [see also (*26*)]. Because these data come from a source independent of the mobility data, they help avoid potential endogeneity issues. Second, the marginal distribution approach is inherently backward looking, capturing only changes that have already occurred. This perspective neglects the forward-looking nature of workers’ decisions. Workers often base their choices on anticipated structural shifts, such as the projected growth of new sectors or the decline of traditional sectors, rather than merely on past trends. Moreover, actual short-term changes in occupational structures may be driven by transient economic fluctuations—such as years of strong or weak job growth—rather than reflecting long-term shifts prompted by technological advances or economic globalization. Because workers’ choices can be shaped by both immediate circumstances and expected future changes, our approach to occupational restructuring incorporates both actual short-term changes in employment numbers and projected long-term occupational shifts.

Our analysis draws on several data sources. To capture occupational restructuring, we first compile information for hundreds of distinct occupations from the BLS’s Occupational Outlook Handbook (OOH), which provides biennial updates on occupational size, growth rates, and other characteristics. These data include current employment estimates and projected changes for the coming decade. We supplement these data with occupation-level education and earnings information from the BLS’s Occupational Employment and Wage Statistics (OEWS) and the Occupational Information Network (O*NET) Production. Last, we merge the resulting OOH-based data with observed worker mobility data from the Current Population Survey Annual Social and Economic Supplement (CPS-ASEC) for the years 2000 to 2020.

We examine how occupational restructuring affects worker mobility through three interrelated analyses: who moves, where they move, and what outcomes they achieve. Our findings show that workers in occupations experiencing immediate contraction have lower mobility rates than those in growing occupations. Also, workers do not necessarily gravitate toward occupations with favorable long-term projections. Rather, those leaving occupations projected to decline often end up in other declining fields instead of switching to occupations expected to grow. Workers in declining occupations are only about one-third as likely to transition to a growing occupation as to another declining occupation, leaving them largely trapped in jobs with limited opportunities and almost half experiencing downward mobility. Below, we present our detailed research findings and then explore their implications for the growing concerns about the future of work and worker mobility amid artificial intelligence and other technological advancements.

## RESULTS

### Occupations with the largest projected job growth and decline

**Fig. 1.**
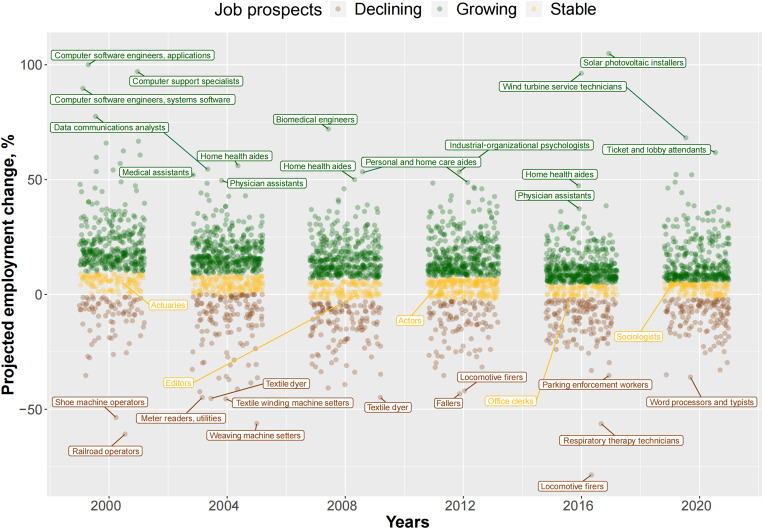
The distribution of occupations by 10-year projected occupational growth rates and year. The figure shows 10-year projected employment change across a selected list of years and highlights the occupations within each year that are projected to have the fastest and slowest growth. For simplicity, data from 2002, 2006, 2010, 2014, and 2018 are omitted. The *y* axis shows the projected job growth in the next 10 years. The definitions of growing, stable, and declining occupations vary from year to year. For a comprehensive overview of the definitions of growing, declining, and stable, please refer to table S1. Data from (*42*–*52*).

We first summarize occupational restructuring in the OOH in Fig. 1, which shows the distribution of projected employment change in various occupations between 2000 and 2020. Using projected employment changes based on the economic outlook and population trends, the BLS classifies occupations into three categories: growing (shown in green), stable (yellow), and declining (chestnut). In the figure, each circle represents one occupation.

The figure shows projections for 3879 occupations between 2000 and 2020. The majority (67.7% or 2394 occupations) were expected to grow in the following 10 years, while a smaller proportion (18.5% or 716 occupations) were expected to decline. Most occupations were projected to change between −15 and 15%. We highlight a few typical occupations in each outlook category. For example, the fastest-growing occupations in the 2000s were those related to computer and information systems, such as software developers, computer support specialists, and information security analysts. Since 2004, health-related occupations, such as biomedical engineers, physician assistants, nursing, psychiatric, and home health aides, have grown notably. In recent years, solar photovoltaic installers, wind turbine service technicians, and other new energy occupations are expected to expand rapidly. Manufacturing occupations, such as textile dyers, textile winding machine setters, and shoe machine operators, experienced an almost 50% projected decline. Railroad operators and locomotive firers, who control and coordinate the operation of trains, are also projected to decline markedly. Many white-collar professions, such as actuaries, editors, and sociologists, were projected to remain stable.

**Fig. 2.**
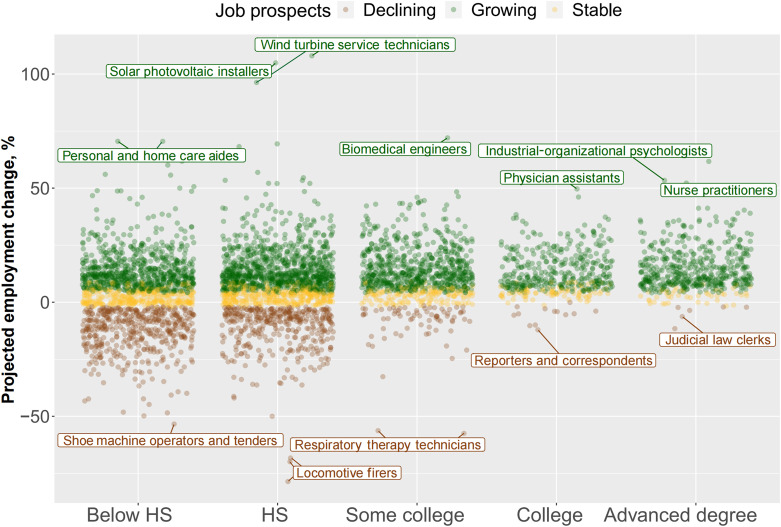
The distribution of occupations by 10-year projected occupational growth rates and occupation-level preferred education. The figure shows 10-year projected employment change across levels of education and highlights the occupations within each educational category that are projected to have the fastest and slowest growth. Data from OOH 2000 to 2020 are combined in this plot. The *x* axis refers to the most common or preferred level of education among workers in each occupation. The education variable is constructed using the required level of education variable in the O*NET database, which estimates the proportions of occupational incumbents in different educational categories. We converted these categories into years of schooling and calculated an average level of required education for each occupation. We then used the following cutoffs to define different education groups: 0 to 11 years (below high school), 12 to 13 years (high school), 14 to 15 years (some college), 16 years (college), and ≥17 years (advanced degree). For a comprehensive overview of the definitions of growing, declining, and stable, please refer to table S1. Data from (*42*–*53*).

Varied occupational prospects are closely related to different educational requirements for occupations. Figure 2 provides a descriptive analysis of predicted growth rates in the next 10 years by the most common level of education among workers in each occupation. Overall, occupations requiring lower levels of education show greater variance in predicted growth rates, with outliers spanning from more than 100% growth in emerging energy sectors to a 75% decline in traditional industries such as locomotive operation. Figure 2 also shows high predicted rates of decline for occupations requiring less education. Far fewer occupations have a declining occupational outlook among occupations preferring high levels of educational attainment. Only four occupations are projected to decline among 373 occupations (1%) that require advanced degrees, whereas 461 of 1487 occupations (31%) in the below high school (HS) group are expected to contract. Health practitioners appear to have distinct occupational outlooks by virtue of their educational credentials. For example, respiratory therapy technician positions, which require some college education, are projected to decline, while nurse practitioner positions, which require master’s degrees, are expected to grow. Overall, occupational restructuring has markedly affected lower-education occupations, which experienced both more extreme growth and risk of decline compared to occupations requiring advanced degrees. We next examine how both immediate employment changes and projected occupational outlooks have affected worker mobility over the past two decades.

### Mobility rates: How structural mobility influences who moves

We first examine the relationship between short-term and long-term occupation-level growth rates and workers’ probabilities of changing occupations. Short-term growth rates indicate changes in employment size between two OOH years, capturing the immediate effects of economic cycles and labor market turbulence. Long-term changes indicate changes projected over the next decade, capturing expected labor market trends such as technological advances, demographic shifts, and economic globalization. We measure the projected change over the next decade using both a continuous projected growth rate variable and a categorical growth variable constructed by the BLS.

**Fig. 3.**
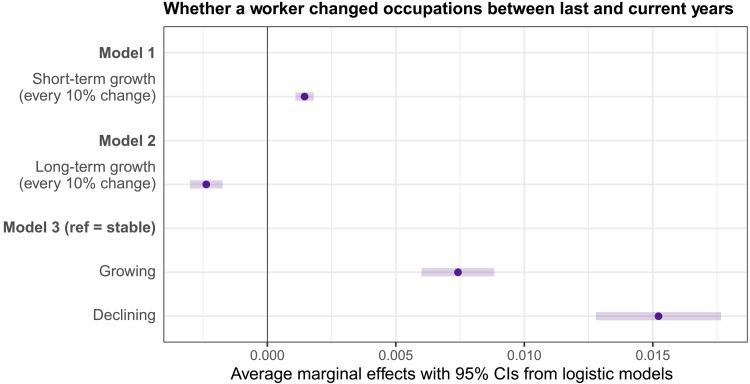
Average marginal effects from logistic regressions predicting workers’ changes between occupations using short- and long-term occupational growth rates and categories. This plot displays the average marginal effects of occupational growth on the probability that a worker changes occupations between the past and current year. The logistic regression results are presented in table S7 (*N* = 921,829 workers). Error bars represent 95% CIs for the average marginal effects. ***P < 0.001 for all coefficients shown. The continuous growth variables capture the effect of a 10% change in growth rates on this probability. Specifically, short-term growth refers to observed occupational employment changes between two OOH years, while long-term growth denotes the projected employment change over the next 10 years.

Figure 3 presents the results of three logistic regression models described in Materials and Methods. In all three models, we include workers’ age, gender, race, ethnicity, and education as controls and year dummies for 2000 to 2020. Model 1 shows a significant association between the occupational growth rate over the past 2 years and workers’ mobility. In particular, workers in occupations experiencing faster growth are slightly more likely to change their occupations. For every 10 percentage point increase in occupational growth, the probability of occupational movement increases by about 0.15 percentage points [95% confidence interval (CI): 0.11 to 0.18], on average. The short-term effect is statistically significant but substantively small. In model 2, we use long-term growth, defined by the projected occupational growth rate over the next 10 years, to predict workers’ mobility. The results indicate that higher projected growth is associated with lower mobility, suggesting that workers in occupations with better long-term prospects are less likely to switch occupations.

A continuous measure of the long-term growth rate may conceal nonlinear relationships. We thus introduce projected occupational growth as a categorical variable in model 3. This model shows a polarization pattern for mobility: Workers in occupations projected to grow and decline are more likely to change their occupations than workers whose occupations are projected to remain stable over the next decade. To simplify our interpretations, we use terms such as more likely (or less likely) to refer to odds when comparing two groups. Specifically, workers in growing occupations are 0.74 percentage points (95% CI: 0.60 to 0.88) more likely to change occupations compared to workers in stable occupations. Workers in declining occupations are 1.52 percentage points (95% CI: 1.28 to 1.77) more likely to move compared to those in stable occupations. In other words, if roughly 10% of workers in stable occupations change occupations each year, then workers in growing occupations have a 10.7% chance of switching, while those in declining occupations have an 11.5% chance.

These results update and provide nuance to DiPrete’s (*27*) earlier findings that occupational contraction forces workers to leave their occupations and find jobs elsewhere. Instead, we find that higher occupational mobility has co-occurred with both occupational contraction and expansion over the past two decades. Our findings also indicate that treating occupational restructuring as a continuous measure, following DiPrete’s approach (*27*), does not capture the separate effects of positive and negative changes in occupational size on workers’ mobility. Because of the lack of data before the 2000s, however, we are unable to detect whether this pattern emerged recently or is a continuing trend.

### Mobility destinations: How structural mobility influences where workers move

We next focus on the occupational destinations of movers in terms of where they move and whether the movement depends on where they come from. Table S3 provides examples of the most common transitions for each transition type. For example, moving from secondary to elementary school teaching represents a transition between two growing occupations. Moving from public administration chief executive to general operations manager illustrates a transition from a declining to a growing occupation. To model mobility, we estimate a series of discrete choice models described in Materials and Methods for workers who report different occupations in the previous and current years in the CPS. These models predict workers’ occupational destinations based on labor market trends, including both short- and long-term occupational growth rates, while controlling for workers’ characteristics and year effects.

In addition, we account for opportunity constraints imposed by the sizes of occupations in the choice set. We assume that the likelihood of workers moving into new occupations is directly related to the availability of job vacancies in these occupations. We use occupational size as a proxy indicator of job vacancies, which are otherwise unobserved in our data. In table S18, we alternatively use BLS projections of job vacancies over the next 10 years as a proxy for job vacancies in the current survey years. The direction of the coefficients is consistent with the main results in table S8, and their magnitudes are, in fact, larger than those reported in table S8.

**Fig. 4.**
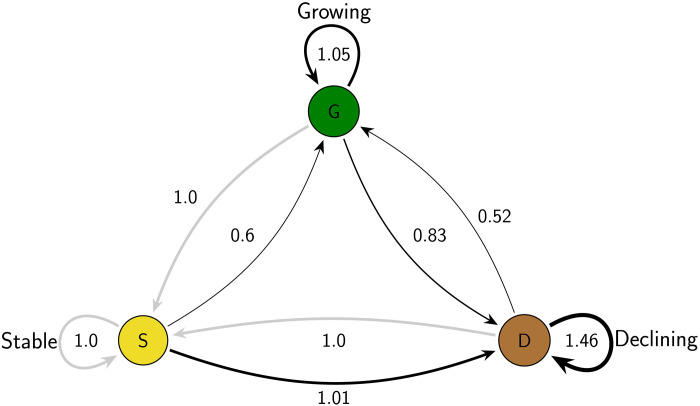
Odds ratios from discrete choice models predicting occupational transitions between different types of projected occupational growth categories. This plot displays odds ratios (ORs) derived from the model estimates presented in table S8, model 4 (*N* = 104,457 workers evaluated across choice sets of occupational alternatives, generating 42,733,727 person-alternative observations). The reference group consists of individuals who transition into stable occupations (indicated by gray lines). This serves as a relative baseline, with all other ORs scaled in comparison to it. For example, among workers coming from a growing occupation, the odds of moving into a growing destination relative to moving into a stable one are 1.05. Similarly, for workers coming from a declining occupation, the odds of moving into a growing occupation relative to moving into a stable one are 0.52. By definition, the odds of transitioning from a growing or declining origin into a stable destination serve as the reference within each origin group and are set to 1.0.

To facilitate interpretation, we plot the results from our model described in Eq. 5 in Materials and Methods (model 4 in table S8) in Fig. 4. The model examines how workers’ transitions between occupations are influenced by both the projected outlook of their current occupation and the projected outlook of other occupations they might potentially move into (their choice set). The numbers in the figure refer to the odds of transitioning between different types of occupational outlook clusters. We treat transitioning into stable occupations as the reference group, and thus, the odds are all equal to 1.

We present our results using odds ratios (ORs) rather than marginal effects because ORs are insensitive to the marginal distribution of the dependent variable and are more reliable when some outcomes are more common than others (*28*), as is the case in our data, where growing occupations are more prevalent. ORs better capture the relative disadvantages faced by workers in declining occupations compared to those in growing or stable ones. Marginal effects are more appropriate than ORs for comparing the “effects” of independent variables across different models (*29*), as shown in Fig. 3. However, in Fig. 4, we use ORs, as all coefficients are estimated from a single model.

The results show two main patterns. First, workers in growing occupations are more likely to transition to other growing occupations than to stable or declining occupations. Specifically, workers originating from a growing occupation have 1.05 times the odds of moving to another growing occupation (versus a stable occupation) but only 0.83 times the odds of transitioning to a declining occupation (versus a stable occupation), all else equal. The reference category consists of transitions into stable occupations (odds = 1). All ORs are calculated within origin groups—that is, comparisons are made only among workers starting in the same type of occupation (growing, stable, or declining). One possible explanation is that growing occupations provide stepping-stone job opportunities or entry portals for workers to transition onto other career paths via “institutional mobility ladders” and “skill transferability” (*30*).

Second, workers in declining occupations often become trapped in these fields—many of which are dead-end jobs—possibly due to limited opportunities for skill-based mobility. Specifically, the odds of moving from declining to growing occupations are less than 36% (0.36 = 0.52/1.46) the odds of moving from declining to declining occupations (with stable occupations as baseline), all else equal. This pattern occurs despite the relatively small share of the population (i.e., under 10%) in declining occupations (see table S5). Overall, the results in Fig. 4 point to a substantial divide in mobility opportunities between workers from growing and declining occupations. Workers from declining occupations tend to have a much lower likelihood of entering growing occupations relative to moving to another declining occupation.

Our results rely on occupational growth categories based on 10-year outlooks rather than short-term growth rates because the BLS defines growing, stable, and declining occupations according to long-term trends rather than short-term fluctuations. As a supplementary analysis, we developed a short-term growth measure based on a ±10% change in employment over 2 years. As shown in table S11 and fig. S7, results are largely consistent with Fig. 4, except that workers in declining occupations are more likely to move to growing occupations than to stable ones. This pattern contrasts with the long-term pattern, which shows a stronger linkage between declining and stable occupations.

### Mobility outcomes: How structural mobility influences upward mobility

As we described above, new job opportunities have emerged at the higher end of the labor market in occupations that offer high and stable earnings and at the lower end in occupations that provide little economic and employment security. To better understand the direction of economic mobility associated with occupational transitions, we analyze occupational growth rates as predictors of moving between low-paying and high-paying occupations. The analyses are restricted to workers who changed occupations over the previous year. Upward mobility is defined by whether the destination occupation’s median earnings are at least 5% higher than the origin occupation’s median earnings. The upward, downward, and horizontal mobility proportions are presented in table S13. We merge the downwardly mobile and immobile groups into one category to simplify the model interpretations. All models include the same workers’ characteristic variables and year dummies. We conduct a robustness check by operationalizing mobility through individual-level rather than occupation-level earnings changes using CPS linked 1-year data (see table S17; most estimates are not statistically significant due to small sample size).

**Fig. 5.**
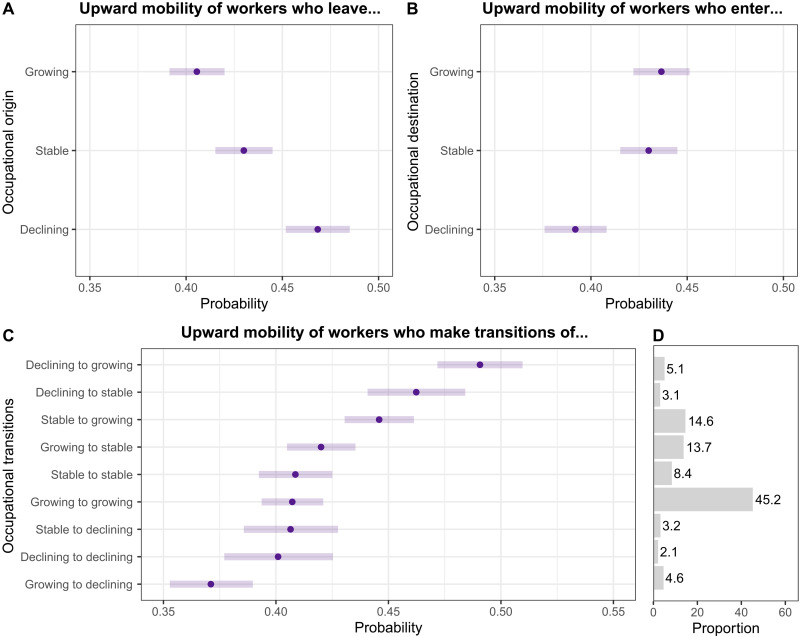
Predicted upward mobility by projected occupational outlook. Predicted probabilities are derived from Models 3–4 in Table S9 (*N* = 96,977 movers). Upward mobility is defined as transitioning to an occupation with median earnings at least 5% higher than those of the origin occupation. All models control for worker characteristics and year fixed effects. Predictions are calculated for non-Hispanic White workers who changed occupations in the past year, have a high school diploma, and were ages 35–56 in 2020. (**A**) Predictions by origin outlook category (occupations workers exit). (**B**) Predictions by destination outlook category (occupations workers enter). (**C**) Predictions by origin–destination combinations of outlook categories. (**D**) Proportion of workers in each origin–destination outlook combination.

We estimate a series of models predicting workers’ upward mobility probabilities using short- and long-term growth rates of origin occupations among workers who changed occupations. Figure 5 presents estimated probabilities for non-Hispanic white workers with a high school degree aged 35 to 56 in 2020. The probability estimates may differ for other demographic groups. We report the original model coefficients in table S9. On average, workers leaving declining occupations have a slightly higher chance of upward mobility (47%; 95% CI: 45.2 to 48.5%) than those leaving stable or growing ones (41%; 95% CI: 39.1 to 42.0%). Entering a declining occupation significantly hinders upward mobility (39% upwardly mobile; 95% CI: 37.6 to 40.8%), with a 5 percentage point lower chance than entering a growing field (44% upwardly mobile; 95% CI: 42.2 to 45.1%). We can further differentiate upward mobility probabilities for workers who made various transitions. Workers transitioning from declining to growing occupations have the highest probability of upward mobility (*P* = 0.49; 95% CI: 47.2 to 51.0%), whereas workers transitioning from growing to declining occupations have the lowest probability (*P* = 0.37; 95% CI: 35.3 to 39.0%). Figure 5D shows that most worker movements occur between growing occupations. Although transitions from declining to growing occupations often lead to upward mobility, they are relatively rare, accounting for only 5.1% of all moves.

## DISCUSSION

Our study proposes occupational growth rates, including both short-term trends and long-term projections in the number of jobs, as indicators of structural mobility. We use administrative data published by the BLS on thousands of detailed occupations over the past two decades. These granular-level data allow us to capture the dynamic nature of job markets, where rapid changes involving job creation, destruction, and transformation occur within specific occupations. Traditional mobility studies often rely on broad occupational categories with high degrees of aggregation, where workers within a large category are all assumed to be relatively homogeneous or at least interchangeable. These studies also largely focus on structural changes within a single occupation, such as agricultural or manual occupations. Our refined approach to linking job changes with microclass occupations reflects uneven structural changes within broad categories shown in Figs. 1 and 2.

By linking structural changes in occupations with individual worker data, we identify several key and sometimes counterintuitive findings. First, contrary to prior research, our findings show no simple relationship between an occupation’s growth and the mobility of its workers. Occupational growth reflects net change—that is, the difference between inflows and outflows—and does not necessarily imply stability for incumbent workers. Our analysis of data from the past two decades suggests that workers in both growing and declining occupations exhibit higher rates of occupational change than those in stable occupations. This pattern implies that occupational mobility has become polarized, with the highest levels of turnover concentrated at both ends of the occupational growth continuum.

Second, we find that workers in growing and declining occupations encounter markedly different mobility opportunities, shaped by asymmetrical structural constraints. Workers in growing occupations have a broader range of mobility pathways, with greater access to transitions into other growing and stable fields. By contrast, workers in declining occupations face more limited options, often resulting in transitions into similar positions within the same declining sector. This pattern challenges the common assumption that workers exiting declining occupations are likely to move into growing fields, where opportunities are presumed to be more abundant. The odds of moving from a declining to a growing occupation are less than 36% of the odds of moving between declining occupations, which indicates a potential trap in low-opportunity occupational segments.

Last, we find that 49.1% (95% CI: 47.2 to 51.0%) of workers transitioning from a declining occupation to a growing occupation experience upward mobility compared to 41% (95% CI: 39.4 to 42.1%) of those moving from one growing occupation to another. However, this higher rate of upward mobility among workers leaving declining occupations should not be interpreted simply as evidence of better opportunities. Because of data limitations, we cannot distinguish between voluntary and involuntary job changes. Prior research indicates that involuntary, displacement-driven transitions are often associated with wage losses. Our data include both workers who proactively and those who involuntarily exit declining occupations. As a result, mobility opportunities may be stratified not only by whether workers exit declining occupations but also by how they leave. Those who are displaced or unable to exit declining occupations may be less likely to experience upward mobility than those who leave voluntarily. Previous research in labor economics has examined the impact of occupational restructuring on worker mobility, primarily from the perspective of displaced workers. Studies consistently show that displaced workers face substantial and persistent earnings losses, especially in declining industries and occupations with limited opportunities for reemployment in similar roles (*31*–*33*). Couch and Placzek (*34*), for example, find that workers from structurally declining sectors such as manufacturing experience worse reemployment prospects and greater earnings losses than those from more stable or growing sectors, largely due to restricted opportunities for comparable employment. While our findings on mobility patterns among workers in declining occupations align with this prior work, we extend beyond discrete displacement events such as layoffs or plant closures to examine all forms of occupational movement. We show that occupational restructuring creates more upward mobility opportunities for workers entering growing occupations. By contrast, workers in declining occupations may experience further marginalization during restructuring due to blocked mobility pathways into growing fields.

Overall, we show that restructuring over the past two decades has created disparities among workers in different occupational groups, driven by the varying growth rates in their respective fields. Workers in declining occupations are increasingly detached from emerging and expanding sectors and opportunities for upward mobility. A possible explanation is that the education, skills, and experience of workers in declining occupations do not align with the qualifications required for job openings in growing occupations, thereby limiting opportunities for career advancement. These differences in training and skill sets result in higher mobility within declining or growing occupational clusters but lower mobility between them. With respect to skill sets, recent research identifies two categories of expanding skills: (i) social-cognitive skills—such as problem-solving, communication, and decision-making—which complement rather than compete with information technology (*35*); and (ii) manual-service skills—such as repair, restoration, and caregiving—which resist automation (*36*). Conversely, skills centered on routine cognitive tasks, standardized manual labor, those typical of middle-income jobs, or math-intensive but socially limited competencies are increasingly in decline (*5*, *35*). Workers in declining occupations often lack higher levels of education, experience in growing fields, and emerging skills, leading to blocked mobility pathways that systematically limit access to opportunities in expanding occupations. This training and skill polarization effectively stratifies mobility opportunities, privileging workers whose expertise aligns with technological and economic transformation while marginalizing those whose skills have become obsolete.

These findings call for future policy development focusing on increasing workers’ ability to respond to the changing work context. Interventions may include, but are not limited to, skill training and upskilling programs, career counseling and guidance services, stronger state investment in higher education to meet workforce needs and lower access barriers, income support for workers shifting jobs or career paths, and other incentives designed to prepare workers for ongoing occupational restructuring. As employment instability and inequality increase, it is crucial to prioritize public investments and policies that support workers in transitioning to growing occupational sectors. This is particularly important for disadvantaged workers in declining fields who may face barriers to entry. These policies should aim to both facilitate movement into expanding fields and ensure job security for those at risk of displacement. These interventions can proactively address the challenges of occupational restructuring and ensure that those most vulnerable to its negative consequences receive the support needed to adapt to and thrive in a changing world of work.

## MATERIALS AND METHODS

### Data

#### Occupational restructuring data

Our primary data source is the OOH, an official career resource featuring detailed information about hundreds of occupations that are designed to assist individuals in making career decisions. Updated biennially, the OOH is developed and maintained by the Office of Occupational Statistics and Employment Projections within the BLS. There were 38 editions from 1949 to 2022; the OOH is now updated continuously (*9*, *37*–*39*). We focus on the years 2000 to 2020, such that all occupations are described using the Census Bureau’s Standard Occupational Classification (SOC). We obtained original administrative data from BLS’s data archives, which were used to prepare the OOHs from 2000 to 2020. OOH employment data sourcing varies by year and occupation but always draws from the BLS’s National Employment Matrix and OEWS. The occupation information includes current and projected employment for occupations over the next 10 years using SOC codes (see the Supplementary Materials for details). Because the OOHs were published biennially, we analyze changes in the size of occupations over these 2-year intervals. We provide examples of occupations described in the OOH in the online supplement. Although the OOH is often described as the BLS’s career guide, we acknowledge that individual workers are unlikely to consult it directly when making career decisions. Instead, workers typically respond to real-time job opportunities and offers, and access to labor market information may vary across demographic groups. Our analysis does not assume that workers rely on the OOH itself. Rather, we treat OOH projections as a proxy for broader expectations or signals about occupational trends that may shape both individual decision-making and societal perceptions of future labor market demand.

#### Worker mobility data

We analyze workers’ mobility data using the IPUMS distribution of the harmonized CPS-ASEC, commonly known as the March CPS, from 2002 to 2020 (*40*). The CPS is a monthly household survey conducted by the Census Bureau for the BLS. The sample contains more than 130,000 individuals and 65,000 households, representing the civilian US population aged 15 and above (excluding individuals in the armed forces, prisons, long-term care hospitals, and nursing homes). Each household is interviewed monthly for four consecutive months during a year and again for the corresponding 4 months a year later. The CPS collects rich information about labor force participation, employment status, unemployment, earnings, work hours, and other characteristics. The data collection includes ASEC, conducted once per year in February, March, and April. The March supplement asks respondents about their main (longest) job in the previous calendar year and thus is suitable for analyzing workers’ mobility over the course of 1 year.

### Measures

We measure occupational restructuring using short- and long-term changes in employment. These two dimensions offer complementary insights into labor market dynamics influencing workers’ occupational changes, each with advantages and disadvantages for assessing structural change. Short-term changes, observed by changes in employment size between two OOH years, capture the immediate effects of economic cycles and labor market turbulence on job displacement or the creation of new opportunities in certain sectors and workers’ decisions to stay in their current positions or seek new jobs. As changes in the Census Bureau’s definitions of occupations may also lead to changes in occupational size estimates, we drop occupations if the changes between 2 years are greater than 400%. Table S6 shows that, at the occupational level, growing occupations had an average growth rate of 2.7% over the past 2 years, whereas declining occupations saw an average decline of 5.7% during the same period.

By contrast, long-term changes projected over the next decade offer an alternative view of the expected evolution of the labor market, shaped by trends such as emerging technological advances, demographic shifts, and economic globalization. We measure the projected change over the next decade using both a continuous projected growth rate variable and a categorical growth variable constructed by the BLS. The original definitions of occupational outlooks provided by the BLS for occupational projections consist of seven categories: growing faster than average, growing as fast as average, growing more slowly than average, little or no change, declining, declining slowly or moderately, and declining rapidly. However, not all of these categories were measured across all years. We thus collapse these definitions into three categories: growing, stable, and declining. Occupations with growth rates substantially above population growth are labeled as growing, while those with slight declines are often categorized as declining. Stable occupations include those with growth rates roughly matching population or labor force growth. This practical approach results in uneven thresholds, especially treating even modest declines as signs of occupational decline (see table S1). table S6 shows that, on average, growing occupations are projected to expand by 15.7% over the next decade, while declining occupations are expected to shrink by 11.8%.

Overall, these long-term growth variables shed light on future demand for various skills and professions, thereby directing workers toward occupations with promising growth prospects or away from those likely to experience decline. The correlation between short- and long-term occupational growth rates is 0.17, which suggests that these two measures capture different aspects of occupational restructuring and do not substantially overlap. Still, workers’ awareness of labor market trends may be incomplete and can vary by factors such as educational attainment or age. Workers who are more aware of such trends may transition earlier than those who are less informed. This information heterogeneity could introduce bias, potentially leading to higher observed mobility rates among more informed workers who make earlier moves.

We conducted two robustness checks to test whether our results hold under different definitions of short- and long-term growth categories. First, we created our own short-term growth measure by classifying occupations as growing or declining if their number of jobs changed by more than 10% over 2 years. The results, shown in tables S10 to S12, are similar to those based on long-term growth categories and confirm the nonlinear relationship between growth rate and worker mobility. Second, we tested a more detailed BLS classification that divides occupations into seven growth categories. Results using these more granular categories, shown in tables S10 to S12, are generally consistent with our main findings. However, we did not observe clear differences within these detailed categories, possibly because some were undefined in certain years.

We construct workers’ mobility using respondents’ jobs in the previous and current years. The CPS-ASEC asked respondents to report their current and longest-term jobs in the prior calendar year. Specifically, question 46 in the ASEC supplement asks respondents whether the longest job they held in the prior calendar year is the same as their current job. If it is not, then question 47 asks for information about that job’s occupation, industry, and class of worker. The occupation information is coded using the 2010 Census occupational classification. Using the 2010 Census codes, we then link the CPS data with the OOH data. Gig and platform work are becoming increasingly important in the US economy, but the CPS data do not systematically capture this form of employment. While some gig-related occupations (e.g., drivers and couriers) are included in occupational codes, the available data often lack the detail needed to distinguish gig work from traditional jobs. The Census Bureau is working to improve measurement in this area, but it remains an evolving field of research (*41*). We discuss possible measurement errors and coding issues in occupational mobility in the online supplement.

As we find that workers in growing and declining occupations have similar rates of moving into unemployment (7.84% versus 7.65%) or leaving the labor force (1.01% versus 0.92%), as described in table S15, we exclude these employment statuses from our main analyses. By linking OOH data with worker data from the CPS, all of our primary analyses are conducted at the individual level. However, analyzing mobility at the occupation level, rather than the individual level, could yield different results because all occupations would be weighted equally rather than weighted by employment size. We provide descriptive statistics of our sample at both the individual and occupation levels in tables S5 and S6. Because we control for individual-level characteristics such as race, gender, and age when modeling mobility, we present our results using individual-level data.

We also construct measures of upward mobility using occupational median earnings in the OEWS data. Among the movers in CPS, we define upward mobility by whether the destination occupation’s median earnings are at least 5% higher than the origin occupation’s median earnings. About 44% of workers experienced upward mobility. Upward mobility indicates that a person moves from a lower-paying to a higher-paying occupation.

### Models

We conduct three sets of analyses. First, we follow DiPrete and Nonnemaker’s (*26*) regression method to examine who moves. Let *P* denote the probability that worker *k* in year *t* changes occupations, and **X** denote the vector of possible individual-level characteristics known to affect occupational mobility rates. We assume that worker *k* is from occupation *i*, where **Z***_i_* denotes occupation-level characteristics for occupation *i*. The parameters α and β refer to the vectors of coefficients for the variables in **Z***_i_* and **X**, including an intercept term. We specify the following equation to estimate the additive effects of structural dynamics and individual-level characteristics on the log odds of the probability of occupational mobility

$$\frac{P}{1-P}=e^{Z_{i}^{\prime }\alpha +X^{\prime }\beta }$$ (1)

where the variables **Z***_i_* include (i) net change rate of occupational size (i.e., the net change in size of occupation *i* from time *t* – 2 to time *t*, divided by the size of occupation *i* in time *t* – 2), (ii) projected growth rate over the next 10 years, and (iii) OOH definitions of occupational growth (i.e., outlook categories). Given that the value of *z_it_* tends to be very small, we adjust the scale of these variables to reflect a 10% change. This transformation enhances the interpretability of the regression coefficients.

Next, we develop a choice set to explain the choices of destinations of those who move—namely, why individuals choose one occupation over another. The discrete choice model predicts the probability that an individual will choose a specific alternative from a set of possible options based on the characteristics of those alternatives and the individual’s attributes. We specify our model as follows. Let *P_ij_* denote the probability that mover *k* in occupation *i* in year *t* chooses a destination occupation *j* out of *J*, with $\sum_{j\in J}P_{\mathrm{ij}}=1$, where *D_j_* is the (employment size) constraints imposed by the destination occupation *j* in year *t*. We assume that it is easier for workers to transition into larger occupations due to the greater availability of vacancies. To account for this, we incorporate a constraint parameter in our model that reflects how the relative size of occupations (measured by the number of incumbents) influences workers’ mobility decisions.

For simplicity, we omit *k* and *t* in our notation below, focusing only on the current occupation *i* and alternative occupation *j* in the choice set. *W_ij_* refers to the workers’ opportunities or preferences that are indicated by either their characteristics or the characteristics of occupations in the choice set. For example, workers’ mobility opportunities are determined by their education and a destination occupation’s growth rate. We denote these vectors as **D***_j_* and **W***_ij_*. We assume that the occupational mobility probability is proportional to the product of constraints (opportunities) from the destination occupations and the workers’ characteristics. The multiplicative assumption specifies the following relationships

$$P_{\mathrm{ij}}\propto \underset{\begin{array}{c} \text{destination}\\ \left(\text{size}\right)\;\text{constraints} \end{array}}{\underbrace{D_{j}}}\cdot \underset{\begin{array}{c} \text{worke}r’s\\ \text{opportunity} \end{array}}{\underbrace{W_{\mathrm{ij}}}}$$ (2)

After normalization,

$$P_{\mathrm{ij}}=\frac{D_{j}\cdot W_{\mathrm{ij}}}{\sum_{j\in J}D_{j}\cdot W_{\mathrm{ij}}}$$ (3)

We model workers’ preferences as a function of workers’ characteristics **X** and characteristics of occupations in the choice set **Z***_ij_*

$$W_{\mathrm{ij}}=\text{exp}\left(Z_{\mathrm{ij}}^{\prime }\gamma +X^{\prime }\delta_{j}\right)$$ (4)

Substituting for **W***_ij_* in Eq. 3 yields the following expression for the choice probability model

$$P_{\mathrm{ij}}=\frac{D_{j}\cdot \text{exp}\left(Z_{\mathrm{ij}}^{\prime }\gamma +X^{\prime }\delta_{j}\right)}{\sum_{j\in J}D_{j}\cdot \text{exp}\left(Z_{\mathrm{ij}}^{\prime }\gamma +X^{\prime }\delta_{j}\right)}$$ (5)

The above equation is the occupational mobility choice model with opportunity constraints. The model is a weighted form of the standard mixed logit model, where utility $\text{exp}\left(Z_{\mathrm{ij}}^{\prime }\gamma +X^{\prime }\delta_{j}\right)$ is weighted by opportunity structure **D***_j_*. In the main analysis, we measure **D***_j_* using the employment size of each occupation. In table S18, we alternatively estimate **D***_j_* using BLS projections of job vacancies for each occupation over the next 10 years. We provide a more detailed discussion of the model in the Supplementary Materials.

Equations 3 and 4 demonstrate that occupational restructuring affects worker mobility through two distinct mechanisms. First, it changes the availability of different jobs (represented by **D***_j_*). This creates limitations regarding where workers can move (opportunity constraints). Second, restructuring alters the attractiveness of different jobs in the available pool (represented by **Z***_ij_*). For instance, some occupations could experience faster growth, leading to higher wages and better job prospects. Conversely, others could become less appealing because of declining labor demands, which can translate to lower wages or fewer opportunities. Changing occupational characteristics also shape worker choice in the job market.

Last, we explore the vertical aspect of mobility by determining the type of occupational movements that result in upward mobility (versus downward mobility or immobility). We categorize downward mobility and mobility to another occupation with similar median earnings as the reference category because these moves generally do not involve positive consequences for workers’ living conditions and are often involuntary. We model whether an individual moves from a lower-paying to a higher-paying job using a binary logistic regression.

## Supplementary Materials

### This PDF file includes:

Supplementary Text Figs. S1 to S7 Tables S1 to S18 References
