# Supplementary material for: Trapped in declining occupations: Barriers to worker mobility in a changing economy
Source: Sci Adv. 2026 Mar 6;12(10):eadx3471. doi: 10.1126/sciadv.adx3471 (PMC12965289; doi:10.1126/sciadv.adx3471)
Supplement: Supplementary file 1 — Supplementary Text Figs. S1 to S7 Tables S1 to S18 References [file sciadv.adx3471_sm.pdf]

Science Advances
AAAS

# Supplementary Materials for

## Trapped in declining occupations: Barriers to worker mobility in a changing economy

Xi Song *et al.*

Corresponding author: Xi Song, xi.song@columbia.edu

*Sci. Adv.* **12**, eadx3471 (2026)
DOI: 10.1126/sciadv.adx3471

**This PDF file includes:**

Supplementary Text
Figs. S1 to S7
Tables S1 to S18
References

# Part A: Occupational Outlook Handbook

The Occupational Outlook Handbook (OOH) is a career resource with detailed job information designed to assist individuals in making decisions about their future work lives. Updated biennially, the OOH has been developed and maintained by the Office of Occupational Statistics and Employment Projections in the Bureau of Labor Statistics. There have been 36 editions so far, with the first publication released in 1949 and the latest released in 2020. The OOH occupations were organized using DOT codes prior to 2000 and O*NET-SOC codes thereafter.

The OOH organizes occupational profiles in a standardized format to facilitate easy comparisons between occupations, although there are slight format variations from year to year. Using the OOH 2020–21 as an example, each profile starts with key information such as median pay, entry-level education, job count, job outlook (growth rate), and employment size changes expected in the next decade. More detailed descriptions include job definitions, typical duties, work environment, work schedules, educational requirements, training, licenses, median pay, and other relevant characteristics. Figure S1 illustrates two pages from the profile of Reporters, Correspondents, and Broadcast News Analysts in the 2019–2029 Edition of OOH. Information used in our analysis includes the 2019 median pay, the number of jobs in 2019, projected employment change between 2019 and 2029, and the job outlook percentage and its classification.

# Reporters, Correspondents, and Broadcast News Analysts

## Summary

**Quick Facts: Reporters, Correspondents, and Broadcast News Analysts**

| | |
|---|---|
| 2019 Median Pay | $46,270 per year<br>$22.25 per hour |
| Typical Entry-Level Education | Bachelor's degree |
| Work Experience in a Related Occupation | None |
| On-the-job Training | None |
| Number of Jobs, 2019 | 52,000 |
| Job Outlook, 2019-29 | -11% (Decline) |
| Employment Change, 2019-29 | -5.800 |

### What Reporters, Correspondents, and Broadcast News Analysts Do

Reporters, correspondents, and broadcast news analysts inform the public about news and events.

### Work Environment

Most reporters and correspondents work for newspaper, website, or periodical publishers or in television or radio broadcasting. Broadcast news analysts mainly work in television and radio.

### How to Become a Reporter, Correspondent, or Broadcast News Analyst

Employers generally prefer workers who have a bachelor's degree in journalism or communications along with an internship or work experience from a college radio or television station or a newspaper.

### Pay

The median annual wage for reporters, correspondents, and broadcast news analysts was $46,270 in May 2019.

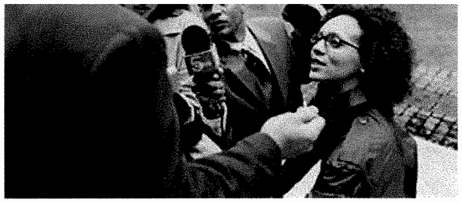

*Journalists need to be persistent in their pursuit of the story as getting the facts of the story can be difficult, especially when those involved refuse to comment.*

in journalism or communications along with an internship or work experience from a college radio or television station or a newspaper.

### Education

Most employers prefer workers who have a bachelor's degree in journalism or communications. However, some employers may hire applicants who have a degree in a related subject, such as English or political science, and relevant work experience.

Bachelor's degree programs in journalism and communications include classes in journalistic ethics and techniques for researching stories and conducting interviews. Some programs may require students to take liberal arts classes, such as history, economics, and political science, so that students are prepared to cover stories on a wide range of subjects. Students may further specialize in the type of journalism they wish to pursue, such as print, broadcast, or multimedia.

Journalism students may benefit from classes in multimedia design, coding, and programming. Because content is increasingly being delivered on television, websites, and mobile devices, reporters need to know how to develop stories with video, audio, data, and graphics.

Some schools offer graduate programs in journalism and communications. These programs prepare students who have a

### Job Outlook

Overall employment of reporters, correspondents, and broadcast news analysts is projected to decline 11 percent from 2019 to 2029. Declining advertising revenue in radio, newspapers, and television will have a negative impact on employment growth for these occupations.

### State & Area Data

Explore resources for employment and wages by state and area for reporters, correspondents, and broadcast news analysts.

## What Reporters, Correspondents, and Broadcast News Analysts Do

Reporters, correspondents, and broadcast news analysts inform the public about news and events happening internationally, nationally, and locally. They report the news for newspapers, magazines, websites, television, and radio.

### Duties

Reporters, correspondents, and broadcast news analysts typically do the following:

- Research topics and stories that an editor or news director has assigned to them
- Investigate new story ideas and pitch ideas to editors
- Interview people who have information, analysis, or opinions about a story or article
- Write articles for newspapers, blogs, or magazines and write scripts to be read on television or radio
- Review articles for accuracy and proper style and grammar
- Develop relationships with experts and contacts who provide tips and leads on stories
- Analyze and interpret information to increase their audiences' understanding of the news
- Update stories as new information becomes available

### Advancement

After gaining more work experience, reporters and correspondents may advance by moving from news organizations in small cities or towns to news organizations in large cities. Larger markets offer job opportunities with higher pay and more responsibility and challenges. Reporters and correspondents also may become editors or news directors.

### Important Qualities

***Communication skills.*** Reporters, correspondents, and broadcast news analysts must be able to report the news. Strong writing skills are important for journalists in all kinds of media.

***Computer skills.*** Journalists should be able to use editing equipment and other broadcast-related devices. They should also be able to use multimedia and coding software in order to publish stories on websites and mobile devices.

***Interpersonal skills.*** To develop contacts and conduct interviews, reporters need to build good relationships with many people. They also need to work well with other journalists, editors, and news directors.

***Persistence.*** Sometimes, getting the facts of a story is difficult, particularly when those involved refuse to be interviewed or to provide comment. Journalists need to be persistent in pursuing the story.

***Stamina.*** The work of journalists is often fast paced and exhausting. Reporters must be able to keep up with the additional hours of work.

## Pay

The median annual wage for reporters, correspondents, and broadcast news analysts was $46,270 in May 2019. The median wage is the wage at which half the workers in an occupation earned more than that amount and half earned less. The lowest 10 percent earned less than $24,520, and the highest 10 percent earned more than $117,170.

[illegible]

### Job Outlook

Overall employment of reporters, correspondents, and broadcast news analysts is projected to decline 11 percent from 2019 to 2029. Declining advertising revenue in radio, newspapers, and television will negatively affect the employment growth for these occupations.

Readership and circulation of newspapers are expected to continue to decline over the next decade. In addition, television and radio stations are increasingly publishing content online and on mobile devices. As a result, news organizations may have more difficulty selling traditional forms of advertising, which is often their primary source of revenue. Some organizations will likely continue to use new forms of advertising or offer paid subscriptions, but these innovations may not make up for lost print-ad revenues.

Declining revenue will force news organizations to downsize and employ fewer journalists. Increasing demand for online news may offset some of the downsizing. However, because online and mobile ad revenue is typically less than print revenue, the growth in digital advertising may not offset the decline in print advertising, circulation, and readership.

News organizations also continue to consolidate and increasingly are sharing resources, staff, and content with other media outlets. For example, reporters are able to gather and report on news for a media outlet that can be published in multiple newspapers owned by the same parent company. As consolidations, mergers, and news sharing continue, the demand for journalists may decrease. However, in some instances, consolidations

### Job Prospects

Reporters, correspondents, and broadcast news analysts are expected to face strong competition for jobs. Those with experience in the field—experience often gained through internships or by working for school newspapers, television stations, or radio stations—should have the best job prospects.

Multimedia journalism experience, including recording and editing video or audio pieces, should also improve job prospects. Because stations and media outlets are increasingly publishing content on multiple media platforms, particularly the web, employers may prefer applicants who have experience in website design and coding.

**Employment projections data for reporters, correspondents, and broadcast news analysts, 2019-29**

| Occupational Title | SOC Code | Employment, 2019 | Projected Employment, 2029 | Change, 2019-29 | |
|---|---|---|---|---|---|
| | | | | Percent | Numeric |
| SOURCE: U.S. Bureau of Labor Statistics, Employment Projections program | | | | | |
| News analysts, reporters, and journalists | 27-3023 | 52,000 | 46,200 | -11 | -5,800 |

### State & Area Data

#### Occupational Employment Statistics (OES)

The Occupational Employment Statistics (OES) program produces employment and wage estimates annually for over 800 occupations. These estimates are available for the nation as a

**Figure S1.** Occupational Profile of Reporters, Correspondents, and Broadcast News Analysts Described in the Occupation Outlook Handbook 2019–2029 Edition (*52*)

## Employment Estimates

Employment estimates for the current year in the OOH predominantly rely on data from the OEWS survey. For example, an estimated 52,000 individuals were employed as reporters, correspondents, and broadcast news analysts in 2019, as shown in Figure S1. The BLS administers the OEWS survey to about 400,000 establishments across all states and industries. The OEWS survey has been fielded as a nationally representative survey since 1996, superseding smaller surveys of establishments at the state and local levels. The OEWS is completed by the owners and management of selected firms. Large firms are categorized by industry according to the North American Industry Classification System (NAICS).

Each NAICS receives a different survey. Those surveys allow the management of large

establishments to specify the number of employees and individual employee wages by occupation. The occupation list in the survey from which employers specify employee counts is establishment-specific. Establishments in different industries are given different potential occupation lists. The length of the occupation list ranges from 50 to 225, varying by the industry of the establishment. Small establishments simply list the occupations of their employees or list the tasks that employees complete on the job, with the BLS coding these tasks into occupations. All establishments specify interval wages for each employee.

Starting in 2002, the OEWS was run biannually, with 200,000 establishments sampled in May and 200,000 establishments sampled in November. Overall, about 1.1 million establishments are sampled over a three-year period. Certain firms are sampled with certainty once every three years, and no establishment is sampled more than once over that time period. Annual OEWS data releases are model-based estimates constructed from the previous three years of data. The OEWS estimates are weighted to be nationally representative. Weights are derived from broad industry and establishment-size groups using the Quarterly Census of Employment and Wages (QCEW) as a reference. The OEWS compares favorably with the Current Population Survey as a source of occupational employment statistics because establishment managers are likely more accurate in assessing employees' occupations than the workers themselves. The survey was not originally designed for time series use but can be adapted for such use with minor modifications (*54*). The OEWS typically has a 20% nonresponse rate and published estimates rely on imputation.

Weighted estimates are calculated by occupation, industry, and geography. Approximately 830 occupations are represented in the OEWS in a given year. Valid employment and wage estimates are available for all 3-digit industries, most 4-digit industries, and selected 5- and 6-digit industries. Estimates for employment and wages are also available for all states and MSAs, nonmetropolitan areas, and territories. The OEWS ignores self-employed workers, owners, and

partners in unincorporated firms, household workers, or unpaid family workers.

## Employment Projections

The OOH publications contain the BLS's National Employment Matrix (NEM), which presents current employment and projected employment in the next ten years for SOC occupations. Data in the NEM are primarily constructed from the establishment-based Occupational Employment and Wage Statistics (OEWS) Survey, which collects employment information of wage and salary workers by occupation and industry except for agricultural and self-employed workers. The NEM data also draw on estimates of the number of self-employed and unpaid family workers in each occupation from the Current Population Survey (CPS). Data from other sources may also be combined with OEWS and CPS to provide estimates of total employment in each OOH occupation. For example, the Office of Personnel Management (OPM) provides employment data on Federal Government workers. Some OOH occupations combine several SOC occupations in the NEM. For these reasons, employment numbers in the OOH are often different from employment data from OEWS, CPS, or other employment surveys.

The exact projected employment growth percent change is not available for OOH before 2008. Between 2002 and 2008, the OOH calculated the job projection using the national employment matrix but only reported descriptive phrases such as "growing much faster than average" or "declining slightly" to describe job outlook. We acknowledge the BLS for sharing the original NEM with us, which allowed us to generate the exact numbers of employment size in the current year and in the next 10 years as well as the projected percentage change. For example, Figure S2 shows the national employment matrix in the OOH for agricultural workers between 2014 and 2024.

**Employment projections data for agricultural workers, 2014–24**

| Occupational Title | SOC Code | Employment, 2014 | Projected Employment, 2024 | Change, 2014–24 | |
|---|---|---|---|---|---|
| | | | | Percent | Numeric |
| Agricultural workers | — | 761,700 | 714,200 | –6 | –47,500 |
| Animal breeders | 45-2021 | 7,000 | 6,900 | –2 | –100 |
| Agricultural equipment operators | 45-2091 | 57,800 | 60,900 | 5 | 3,100 |
| Farmworkers and laborers, crop, nursery, and greenhouse | 45-2092 | 470,200 | 427,300 | –9 | –42,900 |
| Farmworkers, farm, ranch, and aquacultural animals | 45-2093 | 216,100 | 209,100 | –3 | –7,000 |
| Agricultural workers, all other | 45-2099 | 10,600 | 10,000 | –6 | –600 |

**Figure S2.** OOH Employment Projections Data for Agricultural Workers, 2014–24 (*49*)

Below, we describe employment forecast procedures used in the BLS's Occupational Employment Projections Data program. The OOH's typical range for forecasts is ten years, with alternative forecasts with a range of 1 to 13 years appearing occasionally. The Bureau of Labor Statistics (BLS) forecasts require six interrelated steps relying on assumptions and model input from the labor force, aggregated economy, gross domestic product by sector and product, industry output, industry employment, and job openings by occupation (*14*). Figure S3 illustrates these steps. First, the BLS projects the size and demographic composition of the labor force. Second, they use a macroeconomic model to forecast aggregate economic growth. Third, they forecast each industry's final demand. Fourth, input-output relationships estimated from a separate model generate intermediate industry output. Fifth, industry-specific demands are used to estimate industry-specific employment. Finally, using estimated overall industry demand and employment and a matrix translating industry employment to occupational employment, the BLS estimates occupational employment trajectories. Each step is based on separate procedures, models, and related assumptions. BLS analysts approach the six inputs sequentially, as the results produced by each step are key inputs to the following steps and therefore must be reviewed and revised in order. In addition, the sequence may be repeated multiple times to allow feedback and ensure consistency of results. Each step is discussed in more detail below. These steps are summarized from the descriptions of the "Projections Methodology" in the 2018–2019

and 2020–2021 editions of the OOH.

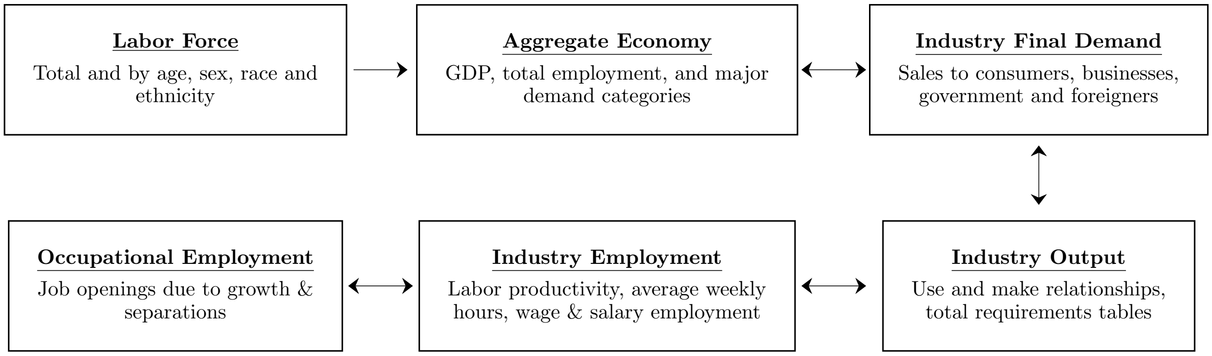


**Figure S3.** BLS's Flow Chart that Illustrates the Six-Step Process of the Projections of Occupational Employment

*Notes*: Double-headed arrows indicate the possibility of repeating certain steps multiple times, enabling feedback between the steps and ensuring consistent estimation. Further information regarding this process can be found in the BLS Handbook of Methods (*55*).

**Step 1: Project Labor Force Size** To arrive at an expected civilian labor force, the BLS first converts resident population projections prepared by the Bureau of the Census to the civilian, non-institutional population. This population projection data by age, gender, race, and ethnic groups are then multiplied by labor participation rate projections of the corresponding demographic group using data from the Current Population Survey (CPS). Finally, groups are added to arrive at a total civilian labor force.

**Step 2: Forecast Aggregate Economic Growth** Before 2012, the BLS relied on the Washington University Macro Model (WUMM) developed by Macroeconomic Advisers (an economic forecasting firm) for its macroeconomic forecasts. The model assumes that consumption follows a life-cycle model and investment is based on a neoclassical model. Foreign sector estimates relied on forecasts from Oxford Economics (an independent economic advisory firm).

Since the 2012–22 forecast, however, the BLS's macroeconomic forecasts have been produced using the MA/US model, licensed from Macroeconomic Advisers by IHS Markit (an information services provider). The assumptions remain the same, but many improvements were made; most notably, the model is explicitly designed to reach a full-employment solution in the target years (*56*). Naive statistical model forecasts estimated during a recession could underestimate the eventual level of occupational employment as the economy approaches full employment.

When using the macro model, the BLS supplies multiple critical variables, such as the in-house labor force projections described above, to the MA/US model as exogenous variables. Other fundamental exogenous variables supplied by BLS include demographic patterns, foreign economic activities, energy prices and availability, and assumptions about fiscal and monetary policy.

**Step 3: Forecast Industry Final Demand** With its macroeconomic model, the BLS forecasts aggregate gross domestic product (GDP) and its distribution by major demand categories. The forecasted GDP sequence serves as a constraint to a projected final demand matrix consisting of demand categories by commodity groups. The data in the "final" commodity demand matrix is summed to the industry level to estimate industry demand. Final adjustments are made at this stage based on the BLS's internal research and analysis by industry experts.

**Step 4: Estimate Industry Output** GDP reflects only sales of final products to final purchasers. Many occupations are devoted entirely to the production of intermediate goods. To derive an industry-level estimate of the intermediate-good output and employment needed to produce a given level of GDP, intermediate and basic industry demands are required. The BLS uses an input-output (I-O) model to convert final demand to intermediate demand. BLS's I-O model consists of a "use" matrix that shows input into the production process, and a "make"

model that shows the commodity output of each industry. Historical relationships and the final demand matrix provide the basis for initial estimates in the I-O table. The BLS reviews and revises the I-O table to account for the changing input and output patterns of each industry. The BLS I-O model is typically used to show how primary inputs, e.g., coal and crude oil, are converted to final products, e.g., total whiteboards purchased in a y ear. For the OOH, the BLS I-O model is inverted. The inverted BLS-I-O model used by the OOH takes in a final GDP from Step 3 and outputs intermediate and basic industry output across all industries.

**Step 5: Project Industry Employment** The next step in the projection process is to obtain the industry employment necessary to produce the projected industry output from the last step. Output by industry is used in regression analysis to estimate worker hours. Next, future output per worker hour is calculated for both salary and wage workers based on studies of trends in productivity and technology. The product of worker hours and productivity, combined with industry output, produces an estimate of employment by industry.

**Step 6: Project Occupational and Self-Employment** Next, an industry-occupation matrix is applied to industry employment estimates to project occupational employment for wage and salary workers in a base year and a projected year (typically ten years in the future). The mapping from industry to occupational employment was called the "National Employment Matrix" starting from 2004 onward. Pre-2004 OOHs used a conceptually similar device to map from industry to occupational employment. Prior to 2004, the BLS Occupational Employment Statistics surveys recorded occupational distributions by industry. The Occupational Employment Statistics surveys were administered every three years. The BLS would forecast how the occupational employment mix would shift for each industry in the future.

For the base year, the matrix is based on occupational staffing patterns, which come from the Census Bureau's Occupational Employment Statistics survey that collects data on a three-year

cycle, as well as self-employment data from CPS. In more recent years' forecasts, data were also taken from the Current Employment Statistics program's Quarterly Census of Employment and Wages (QCEW).

Self-employment was handled differently across years. Prior to the 2012 OOH, self-employed workers' data were estimated using CPS data, separate from the industry-occupation matrix. However, from 2012 onward, the matrix describes the employment of detailed occupations, including those who are self-employed or employed by a private household.

For the projected year, both qualitative and quantitative sources are reviewed by BLS experts to identify structural changes in the economy and approximate changes in the industry-occupation matrix necessary to account for an occupation's share of industry employment. Projected-year employment data for self-employed workers are developed at a less detailed level than wage and salary employment.

**Table S1.** OOH Definitions of a Standard Set of Growth Adjectives

| | | A Standard Set of Occupational Growth Adjectives to Describe the 10-Year Employment Projection | | | | | | | |
|---|---|---|---|---|---|---|---|---|---|
| | | Growing Occupations | | | Stable Occupations | | Declining Occupations | | |
| OOH Publication Year | Projected Employment Change Between | Much faster than average | Faster than average | As fast as average | Slower than average | Little or no change | Decline | Decline slowly or moderately | Decline rapidly |
| 2002 | 2000-2010 | Increase 36 percent or more | Increase 21 to 35 percent | Increase 10 to 20 percent | Increase 3 to 9 perccent | Increase 0 to 2 percent | Decrease 1 percent or more | - | - |
| 2004 | 2002-2012 | Increase 36 percent or more | Increase 21 to 35 percent | Increase 10 to 20 percent | Increase 3 to 9 perccent | Increase 0 to 2 percent | Decrease 1 percent or more | - | - |
| 2006 | 2004-2014 | Increase 27 percent or more | Increase 18 to 26 percent | Increase 9 to 17 percent | Increase 0 to 8 perccent | - | Decrease any amount | - | - |
| 2008 | 2006-2016 | Increase 21 percent or more | Increase 14 to 20 percent | Increase 7 to 13 percent | Increase 3 to 6 perccent | Decrease 2 percent to increase 2 percent | - | Decrease 3 to 9 percent | Decrease 10 percent or more |
| 2010 | 2008-2018 | Increase 20 percent or more | Increase 14 to 19 percent | Increase 7 to 13 percent | Increase 3 to 6 perccent | Decrease 2 percent to increase 2 percent | - | Decrease 3 to 9 percent | Decrease 10 percent or more |
| 2012 | 2010-2020 | Increase 29 percent or more | Increase 20 to 28 percent | Increase 10 to 19 percent | Increase 3 to 9 perccent | Decrease 2 percent to increase 2 percent | - | Decrease 3 to 9 percent | Decrease 10 percent or more |
| 2014 | 2012-2022 | Increase 22 percent or more | Increase 15 to 21 percent | Increase 8 to 14 percent | Increase 3 to 7 perccent | Decrease 2 percent to increase 2 percent | Decrease 3 percent or more | - | - |
| 2016 | 2014-2024 | Increase 14 percent or more | Increase 9 to 13 percent | Increase 5 to 8 percent | Increase 2 to 4 perccent | Decrease 1 percent to increase 1 percent | Decrease 2 percent or more | - | - |
| 2018 | 2016-2026 | Increase 15 percent or more | Increase 10 to 14 percent | Increase 5 to 9 percent | Increase 2 to 4 perccent | Decrease 1 percent to increase 1 percent | Decrease 2 percent or more | - | - |
| 2019 | 2018-2028 | Increase 11 percent or more | Increase 7 to 10 percent | Increase 4 to 6 percent | Increase 2 to 3 perccent | Decrease 1 percent to increase 1 percent | Decrease 2 percent or more | - | - |
| 2020 | 2019-2029 | Increase 8 percent or more | Increase 5 to 7 percent | Increase 3 to 4 percent | Increase 1 to 2 percent | Remain largely unchanged | Decrease 1 percent or more | - | - |
| 2021 | 2020-2030 | Increase 16 percent or more | Increase 11 to 15 percent | Increase 6 to 10 percent | Increase 2 to 5 percent | Decrease 1 percent to increase 1 percent | Decrease 2 percent or more | - | - |
| 2022 | 2021-2031 | Increase 11 percent or more | Increase 8 to 10 percent | Increase 4 to 7 percent | Increase 2 to 3 percent | Decrease 1 percent to increase 1 percent | Decrease 2 percent or more | - | - |
| 2023 | 2022-2032 | Increase 9 percent or more | Increase 5 to 8 percent | Increase 2 to 4 percent | - | Decrease 1 percent to increase 1 percent | Decrease 2 percent or more | - | - |
| 2024 | 2023-2033 | Increase 9 percent or more | Increase 6 to 8 percent | Increase 3 to 5 percent | Increase 1 to 2 percent | Increase less than 1 percent to decrease less than 1 percent | Decrease 1 percent or more | - | - |
| 2025 | 2024-2034 | Increase 7 percent or more | Increase 5 to 6 percent | Increase 3 to 4 percent | Increase 1 to 2 percent | Decrease less than 1 percent to increase less than 1 percent | Decrease 1 percent or more | - | - |

*Notes*: These growth adjectives are used by BLS to compare growth rates among different occupations within the same year. For some years, the growth adjective definitions do not differentiate between decline, decline slowly or moderately, and decline rapidly. The dash sign (-) means that the definitions of these growth adjectives are not available for that year. The employment size and projection data being referred to in the publication were collected two years before the publication year. Data from (*42–52*).

**Table S2.** The Top-10 Fastest Growing and Declining Occupations Based on Projected Percentage Change in the Next 10 Years By OOH Years

| Year | Rank | Fastest Growing | Projected % | Fastest Declining | Projected % |
|---|---|---|---|---|---|
| 2000 | 1 | Computer Software Engineers, Applications | 100.0 | Railroad Operators | -60.8 |
| | 2 | Computer Support Specialists | 97.0 | Shoe Machine Operators and Tenders | -53.6 |
| | 3 | Computer Software Engineers, Systems Software | 89.7 | Telephone Operators | -35.3 |
| | 4 | Network and Computer Systems Administrators | 81.9 | Loan Interviewers and Clerks | -27.6 |
| | 5 | Network Systems and Data Communications Analysts | 77.5 | Motion Picture Projectionists | -27.0 |
| | 6 | Desktop Publishers | 66.7 | Rail-Track Laying and Maintenance Equipment Operators | -26.1 |
| | 7 | Database Administrators | 65.9 | Meter Readers, Utilities | -26.0 |
| | 8 | Personal and Home Care Aides | 62.5 | Farmers and Ranchers | -25.4 |
| | 9 | Computer Specialists, All Other | 60.7 | Radio Mechanics | -24.2 |
| | 10 | Computer Systems Analysts | 59.7 | Communications Equipment Operators, All Other | -21.8 |
| 2002 | 1 | Medical Assistants | 58.9 | Telephone Operators | -56.3 |
| | 2 | Network Systems and Data Communications Analysts | 57.0 | Word Processors and Typists | -38.6 |
| | 3 | Physician Assistants | 48.9 | Textile Knitting and Weaving Machine Setters, Operators, and Tenders | -38.6 |
| | 4 | Social and Human Service Assistants | 48.7 | Sewing Machine Operators | -31.5 |
| | 5 | Home Health Aides | 48.1 | Shuttle Car Operators | -31.3 |
| | 6 | Medical Records and Health Information Technicians | 46.8 | Textile Winding, Twisting, and Drawing Out Machine Setters, Operators, and Tenders | -30.3 |
| | 7 | Physical Therapist Aides | 46.4 | Radio Mechanics | -29.3 |
| | 8 | Computer Software Engineers, Applications | 45.5 | Textile Bleaching and Dyeing Machine Operators and Tenders | -28.7 |
| | 9 | Computer Software Engineers, Systems Software | 45.5 | Roof Bolters, Mining | -27.7 |
| | 10 | Physical Therapist Assistants | 44.6 | Fishers and Related Fishing Workers | -26.8 |
| 2004 | 1 | Home Health Aides | 56.0 | Textile Knitting and Weaving Machine Setters, Operators, and Tenders | -56.2 |
| | 2 | Network Systems and Data Communications Analysts | 54.6 | Textile Winding, Twisting, and Drawing Out Machine Setters, Operators, and Tenders | -45.5 |
| | 3 | Medical Assistants | 52.1 | Textile Bleaching and Dyeing Machine Operators and Tenders | -45.3 |
| | 4 | Physician Assistants | 49.6 | Meter Readers, Utilities | -44.9 |
| | 5 | Computer Software Engineers, Applications | 48.4 | Shuttle Car Operators | -42.4 |
| | 6 | Physical Therapist Assistants | 44.2 | Credit Authorizers, Checkers, and Clerks | -41.2 |
| | 7 | Dental Hygienists | 43.3 | Railroad Brake, Signal, and Switch Operators | -38.5 |
| | 8 | Computer Software Engineers, Systems Software | 43.0 | Mail Clerks and Mail Machine Operators, Except Postal Service | -37.1 |
| | 9 | Dental Assistants | 42.7 | Sewing Machine Operators | -36.5 |
| | 10 | Personal and Home Care Aides | 41.0 | File Clerks | -36.3 |
| 2006 | 1 | Network Systems and Data Communications Analysts | 53.4 | Photographic Processing Machine Operators | -49.8 |
| | 2 | Personal and Home Care Aides | 50.6 | File Clerks | -41.3 |
| | 3 | Home Health Aides | 48.7 | Model Makers, Wood | -40.8 |
| | 4 | Computer Software Engineers, Applications | 44.6 | Patternmakers, Wood | -39.9 |
| | 5 | Veterinary Technologists and Technicians | 41.0 | Telephone Operators | -39.5 |
| | 6 | Personal Financial Advisors | 41.0 | Photographic Process Workers | -36.3 |
| | 7 | Makeup Artists, Theatrical and Performance | 39.8 | Shoe Machine Operators and Tenders | -35.7 |
| | 8 | Medical Assistants | 35.4 | Textile Knitting and Weaving Machine Setters, Operators, and Tenders | -30.9 |
| | 9 | Veterinarians | 35.0 | Coil Winders, Tapers, and Finishers | -30.5 |
| | 10 | Substance Abuse and Behavioral Disorder Counselors | 34.4 | Forging Machine Setters, Operators, and Tenders, Metal and Plastic | -30.4 |

. . . continued

| Year | Rank | Fastest Growing | Projected % | Fastest Declining | Projected % |
|---|---|---|---|---|---|
| 2008 | 1 | Biomedical Engineers | 72.0 | Textile Bleaching and Dyeing Machine Operators and Tenders | -44.8 |
| | 2 | Network Systems and Data Communications Analysts | 53.4 | Textile Winding, Twisting, and Drawing Out Machine Setters, Operators, and Tenders | -40.7 |
| | 3 | Home Health Aides | 50.0 | Textile Knitting and Weaving Machine Setters, Operators, and Tenders | -39.3 |
| | 4 | Personal and Home Care Aides | 46.0 | Shoe Machine Operators and Tenders | -34.8 |
| | 5 | Financial Examiners | 41.2 | Extruding and Forming Machine Setters, Operators, and Tenders, Synthetic and Glass Fibers | -33.9 |
| | 6 | Medical Scientists, Except Epidemiologists | 40.4 | Sewing Machine Operators | -33.7 |
| | 7 | Physician Assistants | 39.0 | Semiconductor Processors | -31.5 |
| | 8 | Skin Care Specialists | 37.9 | Textile Cutting Machine Setters, Operators, and Tenders | -31.0 |
| | 9 | Biochemists and Biophysicists | 37.4 | Postal Service Mail Sorters, Processors, and Processing Machine Operators | -30.3 |
| | 10 | Athletic Trainers | 36.9 | Fabric Menders, Except Garment | -29.8 |
| 2010 | 1 | Personal and Home Care Aides | 70.5 | Shoe Machine Operators and Tenders | -53.4 |
| | 2 | Home Health Aides | 69.4 | Postal Service Mail Sorters, Processors, and Processing Machine Operators | -48.5 |
| | 3 | Biomedical Engineers | 61.7 | Postal Service Clerks | -48.2 |
| | 4 | Helpers–Brickmasons, Blockmasons, Stonemasons, and Tile and Marble Setters | 60.1 | Fabric and Apparel Patternmakers | -35.6 |
| | 5 | Helpers–Carpenters | 55.7 | Postmasters and Mail Superintendents | -27.8 |
| | 6 | Veterinary Technologists and Technicians | 52.0 | Sewing Machine Operators | -25.8 |
| | 7 | Reinforcing Iron and Rebar Workers | 48.6 | Switchboard Operators, Including Answering Service | -23.3 |
| | 8 | Physical Therapist Assistants | 45.7 | Textile Cutting Machine Setters, Operators, and Tenders | -21.8 |
| | 9 | Helpers–Pipelayers, Plumbers, Pipefitters, and Steamfitters | 45.4 | Textile Knitting and Weaving Machine Setters, Operators, and Tenders | -18.2 |
| | 10 | Meeting and Convention Planners | 43.7 | Semiconductor Processors | -17.9 |
| 2012 | 1 | Industrial-Organizational Psychologists | 53.4 | Fallers | -43.3 |
| | 2 | Personal and Home Care Aides | 48.8 | Locomotive Firers | -42.0 |
| | 3 | Home Health Aides | 48.5 | Shoe Machine Operators and Tenders | -35.3 |
| | 4 | Insulation Workers, Mechanical | 46.7 | Postal Service Clerks | -31.8 |
| | 5 | Interpreters and Translators | 46.1 | Log Graders and Scalers | -31.6 |
| | 6 | Diagnostic Medical Sonographers | 46.0 | Postal Service Mail Sorters, Processors, and Processing Machine Operators | -29.8 |
| | 7 | Helpers–Brickmasons, Blockmasons, Stonemasons, and Tile and Marble Setters | 43.0 | Textile Cutting Machine Setters, Operators, and Tenders | -27.1 |
| | 8 | Occupational Therapist Assistants | 42.6 | Semiconductor Processors | -27.1 |
| | 9 | Genetic Counselors | 41.2 | Postal Service Mail Carriers | -26.8 |
| | 10 | Physical Therapist Assistants | 41.0 | Motion Picture Projectionists | -26.5 |
| 2014 | 1 | Wind Turbine Service Technicians | 108.0 | Locomotive Firers | -69.9 |
| | 2 | Occupational Therapist Assistants | 42.7 | Electronic Equipment Installers and Repairers, Motor Vehicles | -50.0 |
| | 3 | Physical Therapist Assistants | 40.6 | Telephone Operators | -42.4 |
| | 4 | Physical Therapist Aides | 39.0 | Postal Service Mail Sorters, Processors, and Processing Machine Operators | -33.7 |
| | 5 | Home Health Aides | 38.1 | Switchboard Operators, Including Answering Service | -32.9 |
| | 6 | Commercial Divers | 36.9 | Photographic Process Workers and Processing Machine Operators | -32.9 |
| | 7 | Nurse Practitioners | 35.2 | Shoe Machine Operators and Tenders | -30.5 |
| | 8 | Physical Therapists | 34.0 | Manufactured Building and Mobile Home Installers | -30.0 |

. . . continued

| Year | Rank | Fastest Growing | Projected % | Fastest Declining | Projected % |
|---|---|---|---|---|---|
| | 9 | Statisticians | 33.8 | Foundry Mold and Coremakers | -27.7 |
| | 10 | Ambulance Drivers and Attendants, Except Emergency Medical Technicians | 33.0 | Sewing Machine Operators | -27.1 |
| 2016 | 1 | Solar Photovoltaic Installers | 104.9 | Locomotive Firers | -78.6 |
| | 2 | Wind Turbine Service Technicians | 96.3 | Respiratory Therapy Technicians | -56.3 |
| | 3 | Home Health Aides | 47.3 | Parking Enforcement Workers | -35.3 |
| | 4 | Personal and Home Care Aides | 38.6 | Word Processors and Typists | -33.1 |
| | 5 | Physician Assistants | 37.3 | Watch Repairers | -29.7 |
| | 6 | Nurse Practitioners | 36.1 | Electronic Equipment Installers and Repairers, Motor Vehicles | -25.6 |
| | 7 | Statisticians | 33.8 | Foundry Mold and Coremakers | -24.0 |
| | 8 | Physical Therapist Assistants | 31.0 | Pourers and Casters, Metal | -23.4 |
| | 9 | Software Developers, Applications | 30.7 | Computer Operators | -22.8 |
| | 10 | Mathematicians | 29.7 | Telephone Operators | -22.6 |
| 2018 | 1 | Solar Photovoltaic Installers | 63.3 | Locomotive Firers | -68.3 |
| | 2 | Wind Turbine Service Technicians | 56.9 | Respiratory Therapy Technicians | -57.5 |
| | 3 | Home Health Aides | 36.6 | Parking Enforcement Workers | -36.7 |
| | 4 | Personal and Home Care Aides | 36.4 | Word Processors and Typists | -33.8 |
| | 5 | Occupational Therapist Assistants | 33.1 | Watch Repairers | -29.6 |
| | 6 | Information Security Analysts | 31.6 | Electronic Equipment Installers and Repairers, Motor Vehicles | -28.6 |
| | 7 | Physician Assistants | 31.1 | Telephone Operators | -28.4 |
| | 8 | Statisticians | 30.7 | Cutters and Trimmers, Hand | -28.4 |
| | 9 | Nurse Practitioners | 28.2 | Postmasters and Mail Superintendents | -27.5 |
| | 10 | Speech-Language Pathologists | 27.3 | Shuttle Car Operators | -25.3 |
| 2020 | 1 | Motion Picture Projectionists | 70.5 | Word Processors and Typists | -36.0 |
| | 2 | Wind Turbine Service Technicians | 68.2 | Parking Enforcement Workers | -35.0 |
| | 3 | Ushers, Lobby Attendants, and Ticket Takers | 61.8 | Nuclear Power Reactor Operators | -32.9 |
| | 4 | Nurse Practitioners | 52.2 | Cutters and Trimmers, Hand | -29.7 |
| | 5 | Solar Photovoltaic Installers | 52.1 | Telephone Operators | -25.4 |
| | 6 | Cooks, Restaurant | 48.9 | Watch Repairers | -24.9 |
| | 7 | Agents and Business Managers of Artists, Performers, and Athletes | 46.3 | Door-To-Door Sales Workers, News and Street Vendors, and Related Workers | -24.1 |
| | 8 | Costume Attendants | 44.3 | Switchboard Operators, Including Answering Service | -22.7 |
| | 9 | Fitness Trainers and Aerobics Instructors | 39.3 | Data Entry Keyers | -22.5 |
| | 10 | Model Makers, Wood | 38.6 | Shoe Machine Operators and Tenders | -21.6 |

# Part B: Occupational Classifications and Crosswalks

## Occupational Classifications in the OOH

The OOH has been widely used by career counselors, urban and local planners, public and private workforce development agencies, policymakers, occupational organizations, individuals, and students engaged in job search because of its reasonably detailed occupational scheme. Each occupation was assigned to one or multiple codes using the Dictionary of Occupational Titles (DOT) between 1949 and 1998, the Occupational Unit (O*NET-OU) in 2000, and the Occupational Information Network Standard Occupational Classification (O*NET-SOC) between 2002 and 2020. Due to the lack of high-quality crosswalks between DOT and OU/O*NET-SOC, we focus on OOH data published after 2002 with a consistent scheme of occupational classification codes based on the Standard Occupational Classification (SOC).

The U.S. government started to develop the SOC in 1966 with the purpose of enhancing comparability in occupational statistics. The first edition was published in 1977, followed by a revision in 1980. The 1980 edition was adopted as part of a tabulation program for the 1980 Census. Since 2000, the system has been updated five times and adopted in all federal programs and demographic surveys (e.g., CPS), including the O*NET. Beginning with O*NET 3.0 published in August 2000, the O*NET adopted an eight-digit O*NET-SOC code by subdividing the six-digit SOC occupation into more detailed occupations with two extended digits. For example, in O*NET-SOC 2010, the SOC 2010 code "11-1011 Chief Executives" was further divided into "11-1011.00 Government Service Executives," "11-1011.02 Private Sector Executives," and "11-1011.03 Chief Sustainability Officers." Thus, starting with the year 2000, the O*NET taxonomic updates are typically referred to as O*NET-SOC #YEAR, e.g. "O*NET-SOC 2009" for the June 2009 release of the O*NET database (O*NET 14.0). The O*NET-SOC 2000 taxonomy was first adopted for use in the 2002 OOH.

The SOC is structured on a four-level system: major group, minor group, broad occupation, and detailed occupation. Each level represents groupings in successively finer detail, which enables users to tabulate or analyze data at different levels of aggregation. Each occupation in the SOC is assigned a 6-digit code. The first two digits represent one of the six major groups, the third digit represents the minor group, the fourth and fifth digits represent the broad occupation, and the sixth digit represents the detailed occupation. For example, "Family and General Practitioners" is coded as 29-1062, with "29" referring to the major group "Healthcare Practitioners and Technical Occupations," "1" referring to the minor group "Health Diagnosing and Treating Practitioners," "06" referring to the broad occupation "Physicians and Surgeons," and the last digit defining the detailed occupation as "Family and General Practitioners." Currently, the SOC includes five editions published in 1977, 1980, 2000, 2010, and 2018. The number of major occupation groups has increased from 21 in the 1977 SOC to 23 in the 2018 SOC, and the number of detailed occupations has changed from 662 in the 1977 edition to 537 in 1980, 822 in 2000, 840 in 2010, and 867 in the 2018 edition.

The O*NET database has been updated 46 times since the year 2000. Starting with the O*NET-SOC 2000, occupational classifications follow an 8-digit format: ##-####.##. The O*NET-SOC 2020 (version 25.0), for instance, divides the SOC occupation into detailed occupation codes "accountants and auditors" (13-2011) into "accountants" (13-2011.01) and "auditors" (13-2011.02). From 2002 to 2008, the OOH utilized 8-digit O*NET-SOC codes. These more detailed occupations can be linked to the SOC by simply dropping the extended digits. From 2010 to 2016, the OOH reported the first six digits of the O*NET code: ##-####. From 2018 onwards, the OOH did not report O*NET-SOC codes for occupations discussed in detail in the main text. We manually assigned these occupations to the appropriate O*NET-SOC codes. From 2018 to 2020, the OOH utilized full 8-digit O*NET-SOC codes for occupations not discussed in detail.

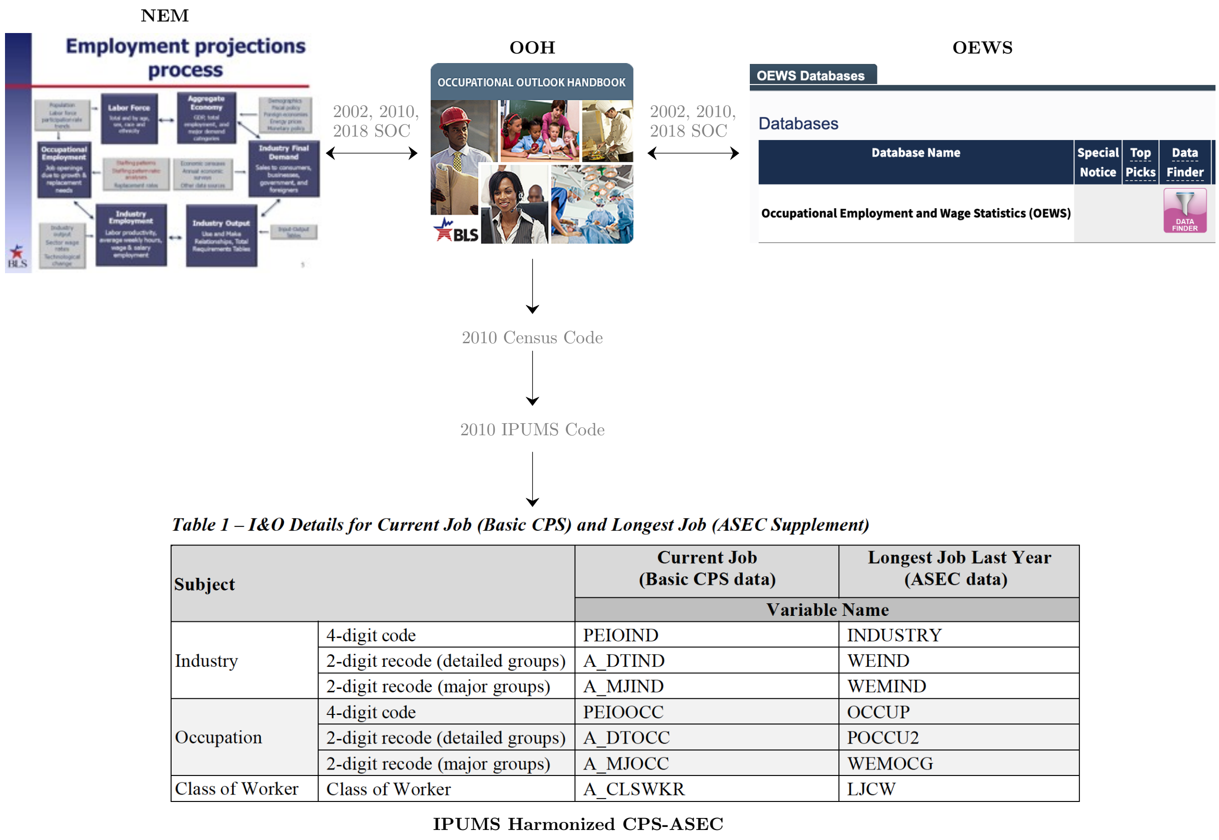


| Subject | | Current Job (Basic CPS data) | Longest Job Last Year (ASEC data) |
|---|---|---|---|
| | | Variable Name | |
| Industry | 4-digit code | PEIOIND | INDUSTRY |
| | 2-digit recode (detailed groups) | A_DTIND | WEIND |
| | 2-digit recode (major groups) | A_MJIND | WEMIND |
| Occupation | 4-digit code | PEIOOCC | OCCUP |
| | 2-digit recode (detailed groups) | A_DTOCC | POCCU2 |
| | 2-digit recode (major groups) | A_MJOCC | WEMOCG |
| Class of Worker | Class of Worker | A_CLSWKR | LJCW |

**Figure S4.** Data Linking Procedures for Occupational Outlook Handbook (OOH), Occupational Employment and Wage Statistics (OEWS), National Employment Matrix (NEM), and the IPUMS Current Population Survey–Annual Social and Economic Supplement (CPS-ASEC)

*Notes*: The published OOH already integrates information from both NEW and OEWS. Our data linkage involves only crosswalking SOC codes in the OOH with the 2010 IPUMS occupation codes used in the CPS-ASEC.

## Crosswalking OOH Occupations to Census Occupations

To merge OOH data with CPS (shown in Figure S4), we need to convert occupational codes in the OOH data with Census occupational codes in CPS. We describe crosswalk procedures below.

**Step (1): Convert O*NET-SOC codes to OOH-SOC codes** O*NET-SOC follows an 8-digit format ##-####.## with the first six-digit referring to the Census Bureau's SOC code. We drop the last two digits after the decimal point of the O*NET-SOC codes to generate SOC codes. This step may generate duplicate cases that need to be removed. For example, Accountants and Auditors include two O*NET-SOC codes 13-2011.01 and 13-2011.02 but only one SOC code 13-2011.

**Step (2): Generate SOC2002, SOC2010, and SOC2018 using OOH-SOC codes** SOC codes have been updated in 2000, 2010, and 2018. The OOH-SOC codes have mixed SOC codes from these three different versions. We attach SOC2002, SOC2010, and SOC2018 codes and titles to the file saved in the last step using the Census Bureau's 2002, 2010, and 2018 occupation code lists.

**Step (3): Impute missing SOC2002, SOC2010, and SOC2018** Some SOC occupation codes and titles cannot be attached to OOH-SOC codes in the file from the last step because of the last digit. For example, OOH 2002 includes a SOC code 13-1031 ("Claims Adjusters, Appraisers, Examiners, and Investigators"), whereas the Census Bureau's 2002 occupation code list only has 13-1030 ("Claims Adjusters, Appraisers, Examiners, and Investigators"). We then treat 13-1031 the same as 13-1030.

**Step (4): Impute missing SOC2010 using SOC2002 and SOC2018** Some SOC occupations with valid SOC2002 and SOC2018 Codes may have missing SOC 2010 Codes. We use cross-walk files prepared by the Census Bureau to update missing SOC2010 codes with non-missing SOC2002 and SOC2018 codes.

**Step (5): Convert SOC2010 to Census 2010 Codes** The U.S. Census Bureau has also developed its occupational classification scheme based on detailed SOC occupations or aggregations of SOC occupations. The SOC codes are similar to, but not exactly the same as, the occupational codes used by the Census Bureau. We merge Census 2010 codes into the OOH-SOC data file using the Census Bureau's "2010-occ-codes-with-crosswalk-from-2002-2011" file. This step generates two new variables CEN2010 and CEN2010_title.

**Step (6): Convert Census 2010 codes to IPUMS-modified Census 2010 codes** The IPUMS CPS project harmonizes microdata from the Current Population Survey (CPS). Occupations are coded using IPUMS 2010 Codes (OCC2010), a harmonized occupation coding scheme based on the Census Bureau's 2010 occupation classification scheme (CEN2010). However, there are some cases where OCC2010 collapses detailed occupations in CEN2010 to facilitate comparability over time. The occupational titles for some OCC2010 codes thus may not correspond to the 2010 occupation code labels. For example, the OCC2010 combines the original CEN2010 codes of 4700 ("First-Line Supervisors of Non-Retail Sales Workers") and 4710 ("First-Line Supervisors of Retail Sales Workers) into the OCC2010 code of 4700 ("First-Line Supervisors of Sales Workers"). Following instructions on the IPUMS website (`https://forum.ipums.org/t/occ2010-really-2010-census-occupations/3792`, accessed on 8-14-2025), we created a crosswalk of the official 2010 coding scheme (CEN2010) and the codes used in OCC2010. Specifically, we downloaded 2011-2019 IPUMS-CPS data with variables OCC (i.e., CEN2010) and OCC2010, which show the mapping of the 2010 occupation classification scheme to the IPUMS variable OCC2010. The final crosswalk can be found in the Excel file ("crosswalk_CPS_OOH2002_2020.xlsx") on the project website.

# Part C: Measuring Workers' Occupational Mobility Using the Current Population Survey

## Data Description

Our mobility analyses draw on data from the IPUMS Current Population Survey Annual Social and Economic Supplement (CPS-ASEC) from 2002 to 2020 (*57*). The data have been widely used in previous studies on workers' occupational mobility (*58–60*). IPUMS-CPS provides harmonized microdata from the Current Population Survey (CPS), which is a monthly U.S. household survey conducted by the U.S. Bureau of Labor Statistics and the Census Bureau. The sample contains about 60,000 occupied households each month and is a nationally representative sample of people aged 15 and above in the civilian U.S. population (excluding those in the Armed Forces, prisons, long-term care hospitals, and nursing homes). Each household is interviewed monthly for four consecutive months during a year, and again for the corresponding four months a year later. The CPS collects rich information about labor force participation, employment status, unemployment, earnings, hours of work, and other characteristics. The data collection includes a few Annual Social and Economic Supplements (CPS-ASEC), which are conducted once a year in the months of February, March, and April. In particular, the March supplement asks respondents about their main (longest) job in the previous calendar year and thus allows for an analysis of workers' mobility over a one-year period. We choose 2000 as the beginning year because occupations in earlier years of CPS cannot be reliably linked with OOH. Specifically, before 2000, OOH occupations were coded using the Dictionary of Occupational Titles (DOT) or Occupation Unit (OU), rather than the Standard Occupational Classification (SOC) codes.

**Table S3.** The Most Common Types of Occupational Transitions by Occupational Outlooks of Origin and Destination Occupations in 2020

| | | Most Common Types of Occupational Transitions | |
|---|---|---|---|
| Origin | Destination | From | To |
| Growing | Growing | Secondary School Teachers | Elementary and Middle School Teachers |
| Growing | Stable | Maids and Housekeeping Cleaners | Janitors and Building Cleaners |
| Growing | Declining | Other Production Workers Including Semiconductor Processors and Cooling and Freezing Equipment Operators | Managers, NEC. (including Postmasters) |
| Stable | Growing | Cleaners of Vehicles and Equipment | Driver/Sales Workers and Truck Drivers |
| Stable | Stable | Sales Representatives, Wholesale and Manufacturing | Retail Salespersons |
| Stable | Declining | Retail Salespersons | Cashiers |
| Declining | Growing | Chief Executives and Legislators/Public Administration | General and Operations Managers |
| Declining | Stable | Bookkeeping, Accounting, and Auditing Clerks | Retail Salespersons, and Customer Service Representatives |
| Declining | Declining | Office Clerks, General | Secretaries and Administrative Assistants |

*Notes*: The table shows the most common types of workers' occupational transitions between two consecutive years in CPS. Occupational movements into unemployment or out of the labor force are excluded. The sample is restricted to workers aged 16–65. Data from (*52, 61*).

## Coding Issues

Kambourov and Manovskii (*62*) discussed several possible sources of biases in mobility estimates using the March CPS. In the first month and the fifth month since they participated in the CPS survey, respondents are asked to describe their main job activities (e.g., tasks and duties). Based on these descriptions, coders then assign Census occupational codes to respondents. During the second to the fourth month and the sixth to the eighth month, occupations are coded according to a dependent coding technique. Specifically, respondents were asked about (1) whether they have changed companies; (2) whether their usual work activities and duties have changed since last month; and (3) whether the reporting of work performed and job activities from the previous month is still accurate. These dependent coding procedures tend to dramatically reduce observed occupational mobility rates.

However, (*62*) note that even when adopting dependent coding, a notable number of coding errors may persist. Only individuals who meet specific criteria, such as remaining in the same company, not changing work activities and duties, and providing accurate reporting of their work performed in the previous month, are assigned the same occupation code as the previous month. Individuals who changed companies but not occupations, as well as those who changed work activities and duties, are coded independently. Moreover, many individuals who reported inaccuracies in the job description for the previous month are also coded independently. One possible reason is that individuals often complete CPS surveys on behalf of other household members, which can lead to inaccuracies. Consequently, a substantial proportion of the CPS sample is coded independently, potentially resulting in an overestimation of occupational mobility rates.

To avoid this problem, we decide not to link the monthly CPS data across waves to measure workers' mobility. Instead, we use the Annual Social and Economic Supplement (ASEC) of the CPS, which has adopted a dependent coding procedure since 1970. Question 46 in the ASEC supplement asks respondents whether the longest job they held in the prior calendar year is the same as their current job. If it is not, question 47 further asks what that job was, as well as information about the occupation, industry, and class of worker. These survey answers are coded into supplement variables that define the longest job in the previous year.

# Part D: Estimating Discrete Choice Models with Occupational Size Constraints

We model individuals' occupational choices in the framework of discrete choice models, also known as conditional logit models, multinomial logit models, or generalized mixed multinomial logit models. These models are often used to represent decision-making behaviors where individuals select one or more options from a given set of alternatives, usually assuming that they choose the option(s) with the highest utility. The model was first proposed by McFadden (*63–65*) and later elaborated in a few other works (*66–68*). Bruch and collaborators (*69, 70*) provide reviews of applications of the model in sociological research.

In this section, we provide an overview of standard discrete choice models and then delve into the specific adaptations necessary for studying occupational mobility. Previous studies have employed these models to analyze decisions related to various locations such as neighborhoods, school districts, travel destinations, and other potential outcomes. When studying occupational mobility, the choice set typically includes occupations classified by SOC or Census codes. The main emphasis lies in examining the specific occupation chosen from the available alternatives.

We consider discrete choice models with a single choice (i.e., a single destination). The data structure for estimating such models is illustrated in Table S4. Each individual ($k$) currently in occupation $i$ has multiple lines of data ($J$) representing potential destination alternatives. Each line represents a "person-alternative," and the set of $J$ alternatives constitutes the individual's choice set. For illustration purposes, we assume a choice set size of $J = 1,000$ for all individuals. Individual characteristics ($X_k$) remain constant within individuals (e.g., education), while features of the alternative options ($Z_{ij}$), such as occupational outlook and average earnings, can vary within individuals. We reshaped our CPS individual-level mobility data to a discrete choice format. The original data contain 4,462,254 individuals and 454 possible Census occu-

pations in the choice set. After the reshaping, the sample for the discrete choice model contains 454*4,462,254 person-alternative choice observations. We then excluded data from odd years and with missing information on occupational characteristics. The final size of the analytical sample is 412,710,068.

**Table S4.** Data Structure for Estimating Discrete Choice Models

| Person ID | Origin Occupation | Alternative Occupations | Destination Occupation | Origin Outlook | Alternative Outlook | Origin Earnings | Alternative Earnings | Education | Choice |
|---|---|---|---|---|---|---|---|---|---|
| 1 | 200 | 1 | 210 | Growing | Growing | 60000 | 80000 | HS | 0 |
| 1 | 200 | 2 | 210 | Growing | Growing | 60000 | 70000 | HS | 0 |
| ... | ... | ... | ... | ... | ... | ... | ... | ... | ... |
| 1 | 200 | 210 | 210 | Growing | Stable | 60000 | 65000 | HS | 1 |
| ... | ... | ... | ... | ... | ... | ... | ... | ... | ... |
| 1 | 200 | 1000 | 210 | Growing | Declining | 60000 | 40000 | HS | 0 |
| 2 | 200 | 1 | 530 | Declining | Growing | 35000 | 80000 | Below HS | 0 |
| 2 | 520 | 2 | 530 | Declining | Growing | 35000 | 70000 | Below HS | 0 |
| ... | ... | ... | ... | ... | ... | ... | ... | ... | ... |
| 2 | 520 | 530 | 530 | Declining | Stable | 35000 | 35000 | Below HS | 1 |
| ... | ... | ... | ... | ... | ... | ... | ... | ... | ... |
| 2 | 520 | 1000 | 530 | Declining | Declining | 35000 | 40000 | Below HS | 0 |

In standard discrete choice models, the outcome $Y_{kij}$ is an indicator variable denoting which occupation (indexed by $j$) is chosen by the $k$th individual currently in occupation $i$ ($k = 1, ..., K; i = 1, ..., I; j = 1, ..., J$). Let $U_{kij}$ be the latent utility or preference that the $k$th individual chooses the $j$th occupation. Let $P_{kij}$ denote the probability that individual $k$ currently in occupation $i$ chooses a destination occupation $j$ out of $J$, with $\sum_{j \in J} P_{kij} = 1$. The utility of an occupation for an individual depends on occupation characteristics, possibly interacting with individuals' sociodemographic characteristics. Occupation characteristics, denoted by $\mathbf{Z}_{ij}$, include occupation outlook and average earnings. Individual characteristics denoted by $\mathbf{X}_k$ include fixed demographic characteristics such as age, sex, race, ethnicity, and education. Let $\phi_{kij}$ represent the contribution of unobserved characteristics of individuals and potential

occupations to utility. Individuals' preference is thus represented as

$$U_{kij} = F(\mathbf{Z}_{ij}, \mathbf{X}_k, \phi_{kij}) \tag{6}$$

When individuals choose where to move, they implicitly compare occupations in their choice set—that is, occupations that they prefer and where they may move with a nonzero probability. The difference in utility between the $j$th and the $l$th occupation is $U_{kij} - U_{kil}$, which is a function of observed and unobserved characteristics of individuals and different occupations.

The estimation of discrete choice models typically assumes that the unobserved characteristics $\phi_{kij}$ follow a type I extreme value (Gumbel) distribution, so that

$$\mathbf{P}_{kij} = \frac{\exp(\mathbf{Z}_{ij}\gamma + \mathbf{X}_k\beta_j)}{\sum_{j\in J}\exp(\mathbf{Z}_{ij}\gamma + \mathbf{X}_k\beta_j)} \tag{7}$$

A potential limitation of the discrete choice model in the case of occupational choices is that the size of each alternative or vacancies in each occupation is ignored or implicitly assumed to be irrelevant to the mobility process. However, declining or growing job opportunities across occupations can substantially impact individuals' mobility decisions and outcomes. Therefore, accounting for occupational employment size constraints becomes crucial in understanding occupational choices. To address this issue, we introduce the size constraints imposed by the destination occupation $j$, $\mathbf{D}_{kj}$ to the standard discrete choice model. Assume that the occupational mobility probability is proportional to the *product* of constraints (opportunities) from the destination occupations and workers' characteristics. The multiplicative assumption specifies the following relationships:

$$\mathbf{P}_{kij} \propto \underbrace{\mathbf{D}_{kj}}_{\substack{\text{destination}\\\text{(size) constraints}}} \cdot \underbrace{\mathbf{U}_{kij}}_{\substack{\text{worker's}\\\text{opportunity or utility}}} \tag{8}$$

We then modify the occupational choice probability in equation (7) as follows,

$$\mathbf{P}_{kij} = \frac{\mathbf{D}_{kj} \cdot \exp(\mathbf{Z}_{ij}\gamma + \mathbf{X}_k\beta_j)}{\sum_{j \in J} \mathbf{D}_{kj} \cdot \exp(\mathbf{Z}_{ij}\gamma + \mathbf{X}_k\beta_j)} \tag{9}$$

We call the above equation the occupational mobility choice model with opportunity constraints. The model is a weighted form of the standard mixed logit model, where utility $\exp(\mathbf{Z}_{ij}\gamma+\mathbf{X}_k\beta_j)$ is weighted by opportunity structure $\mathbf{D}_{kj}$. The model can be estimated using programs for mixed logit models with $\log(\mathbf{D}_{kj})$ as offset variables, whose coefficients are fixed at 1. In the main analysis, we use the employment size of each occupation as a proxy measure of $\mathbf{D}j$. In *SM* Table S18, we alternatively estimate $\mathbf{D}j$ using BLS projections of job vacancies for each occupation over the next ten years.

Given that the occupational choice set encompasses all Census occupational categories, the number of observations in the discrete choice model becomes exceedingly large. Consequently, the computation of choice probabilities for each person-alternative observation becomes burdensome. A common solution is to draw a sample from the person-alternative observations within each respondent (*69, 71*). In other words, it is possible to derive consistent estimates of the discrete choice model by using all information on actually chosen alternatives and a random subsample of unchosen alternatives. Yet, researchers still face the challenge of determining the probability of sampling the $j$th destination for the $k$th individual, commonly represented as $q_{kij}$. In practice, there are no definite guidelines available for selecting a specific value of $q_{kij}$. To avoid this issue, we run our models on a high-performance computing cluster for the full sample. For replication purposes, we provide a subsample of our data that includes all chosen alternatives with probability $q_{kij} = 1.0$ and unchosen alternatives with probability $q_{kij} = 0.1$. This subsample can be downloaded from the project website.

# Part E: Main Analyses

**Table S5.** Descriptive Statistics of Occupational and Demographic Characteristics of Workers in the Current Population Survey

| | All Workers | Stayers | Movers |
|---|---|---|---|
| | A. Occupational Characteristics | | |
| **Occupational Growth Rate Over the Last Two Years, %** | 0.56 | 0.48 | 1.15 |
| | (17.68) | (17.57) | (17.36) |
| **Projected Occupational Growth Rate Over the Next Decade, %** | 10.01 | 10.07 | 9.50 |
| | (10.41) | (10.39) | (10.41) |
| **Projected Occupational Outlook Categories, %** | | | |
| Growing | 63.81 | 63.76 | 64.20 |
| Stable | 27.29 | 27.57 | 25.57 |
| Declining | 8.90 | 8.67 | 10.23 |
| **Occupation-Level Earnings in 2020 Dollars** | 56,671.97 | 58,098.02 | 52,058.85 |
| | (32,015.55) | (32,629.85) | (28,441.31) |
| | B. Demographic Characteristics | | |
| **Age Group, %** | | | |
| 15–35 | 38.84 | 36.02 | 50.85 |
| 36–56 | 48.28 | 50.85 | 39.78 |
| 56–65 | 12.88 | 13.13 | 9.37 |
| **Gender, %** | | | |
| Male | 51.39 | 51.98 | 51.50 |
| Female | 48.61 | 48.02 | 48.50 |
| **Race, %** | | | |
| White | 80.69 | 81.10 | 78.03 |
| Black | 10.66 | 10.28 | 13.52 |
| Asian and Others | 8.65 | 8.62 | 8.45 |
| **Hispanics, %** | | | |
| Yes | 16.39 | 16.56 | 15.62 |
| No | 83.61 | 83.44 | 84.38 |
| **Levels of Education, %** | | | |
| Below High School | 11.85 | 10.90 | 12.62 |
| High School | 28.44 | 28.45 | 30.09 |
| Some College | 29.39 | 28.89 | 31.80 |
| BA and Above | 30.31 | 31.76 | 25.49 |
| *N* | 978,635 | 824,603 | 97,226 |

*Notes*: Numbers in parentheses are standard deviations for continuous variables. All workers include workers who did not change occupations (stayers), those who changed occupations (movers), and those who became un-

employed or exited the labor force. Movers refer to workers who reported occupational changes between the last and current calendar years in CPS-ASEC. The occupational growth rate over the last two years is defined as the percent employment change between two OOH years. The projected occupational growth rate over the next decade is defined as the projected percent change in employment over the projection's decade. The OOH crosswalks between projected occupational growth rate and growth categories (growing, stable, and declining occupations) are included in *SM* Table S1. The size of some occupations changed dramatically between two OOH years because of changes in the Census codes. We dropped occupations that increased more than 400% in two years, which account for about 1.4% of all cases in the CPS sample. Data from (*61*).

**Table S6.** Descriptive Statistics of Occupation-Level Characteristics from the Current Population Survey

| | Occupational Characteristics | | |
|---|---|---|---|
| | **Growing** | **Stable** | **Declining** |
| **Occupational Growth Rate Over the Last Two Years, %** | 2.72 | -1.42 | -5.73 |
| | (20.09) | (18.34) | (15.02) |
| **Projected Occupational Growth Rate Over the Next Decade, %** | 15.66 | 3.68 | -11.75 |
| | (8.62) | (3.07) | (9.40) |
| **Current Employment Size** | 385,643 | 306,701 | 179,969 |
| | (614,764) | (590,867) | (429,122) |
| **Projected Employment Size** | 444,655 | 318,341 | 164,312 |
| | (705,055) | (613,266) | (406,098) |
| **Occupation-Level Earnings in 2020 Dollars** | 52,016.48 | 46,717.99 | 37,582.52 |
| | (26,991.23) | (24,401.61) | (15,819.27) |
| **Number of Occupation-Years** | 2,693 | 1,273 | 821 |

*Notes*: Numbers in parentheses are standard deviations for continuous variables. The occupational growth rate over the last two years is defined as the percent employment change between two OOH years. The projected occupational growth rate over the next decade is defined as the projected percent change in employment over the projection's decade. The OOH crosswalks between projected occupational growth rate and growth categories (growing, stable, and declining occupations) are included in *SM* Table S1. The size of some occupations changed dramatically between two OOH years because of changes in the Census codes. We dropped occupations that increased more than 400% in two years, which account for about 9% of all occupation-year observations in the sample. Data from (*42–52*).

**Table S7.** Coefficients from Logistic Regression Models Predicting Occupational Changes Using Occupation-Level and Workers' Characteristics

| | Whether a Worker Changed Occupations Between Last and Current Calendar Years | | |
|---|---|---|---|
| | (1) | (2) | (3) |
| **Occupational Growth Rate Over the Last Two Years** | 0.016*** | | |
| | (0.002) | | |
| **Projected Occupational Growth Rate Over the Next Decade** | | -0.026*** | |
| | | (0.003) | |
| **Projected Occupational Outlook Categories (ref: Stable)** | | | |
| Growing | | | 0.081*** |
| | | | (0.008) |
| Declining | | | 0.162*** |
| | | | (0.013) |
| **Workers' Characteristics** | Yes | Yes | Yes |
| **Year Dummies** | Yes | Yes | Yes |
| **Number of Observations** | 921,829 | 921,829 | 921,829 |

*Notes*: The occupational growth rates over the last two years and over the next decades are quantified by increments of 10 percent. All models include workers' characteristics and year dummies as controls. Workers' characteristics include age, gender, race, ethnicity, and education.
$^{*}p < 0.05$; $^{**}p < 0.01$; $^{***}p < 0.001$; two-tailed tests.

**Table S8.** Coefficients from Discrete Choice Models Predicting Occupational Destinations Using Occupation-Level and Workers' Characteristics

| | Whether a Worker Moved into an Occupation in the Choice Set | | | |
|---|---|---|---|---|
| | (1) | (2) | (3) | (4) |
| **Occ. Dest. Growth Rate Over the Last Two Years** | -0.163*** | | | |
| | (0.012) | | | |
| **Occ. Dest. Projected Growth Over the Next Decade** | | -0.079*** | | |
| | | (0.016) | | |
| **Occ. Dest. Projected Outlook Category (ref: Stable)** | | | | |
| Growing | | | -0.243*** | -0.516*** |
| | | | (0.040) | (0.055) |
| Declining | | | 0.046 | 0.008 |
| | | | (0.054) | (0.058) |
| **Occ. Origin** $t$ *** Occ. Dest.** $t+1$ | | | | |
| Growing * Growing | | | | 0.560*** |
| | | | | (0.017) |
| Growing * Declining | | | | -0.200*** |
| | | | | (0.028) |
| Declining * Growing | | | | -0.145*** |
| | | | | (0.028) |
| Declining * Declining | | | | 0.370*** |
| | | | | (0.036) |
| **Workers' Characteristics** | Yes | Yes | Yes | Yes |
| **Year Dummies** | Yes | Yes | Yes | Yes |
| **Occupational Size Constaint** | Yes | Yes | Yes | Yes |
| **Number of Workers** | 104,457 | 104,457 | 104,457 | 104,457 |
| **Number of Observations** | 42,733,727 | 42,733,727 | 42,733,727 | 42,733,727 |

*Notes*: The discrete choice models are described in equations (7)-(9). The occupational growth rates over the last two years and over the next decades are quantified by increments of 10 percent. All models control for workers' characteristics and year dummies, which are added as interactions between these variables and the characteristics of occupations in the choice set. Workers' characteristics include age, gender, race, ethnicity, and education.
$^{*}p < 0.05$; $^{**}p < 0.01$; $^{***}p < 0.001$; two-tailed tests.

**Table S9.** Coefficients from Logistic Regression Models Predicting Upward Mobility Using Occupation-Level and Workers' Characteristics

| | Whether a Mover Experienced Upward Mobility (5% or More Increase in Occupation-Level Earnings) | | | |
|---|---|---|---|---|
| | (1) | (2) | (3) | (4) |
| **Occ. Origin Growth Rate Over the Last Two Years** | -0.0007** | | | |
| | (0.0002) | | | |
| **Occ. Dest. Growth Rate Over the Last Two Years** | 0.0008** | | | |
| | (0.0002) | | | |
| **Occ. Origin Projected Growth Rate Over the Next Decade** | | 0.004 | | |
| | | (0.007) | | |
| **Occ. Dest. Projected Growth Rate Over the Next Decade** | | 0.033*** | | |
| | | (0.007) | | |
| **Occ. Origin Projected Outlook Categories (ref: Stable)** | | | | |
| Growing | | | -0.100*** | 0.047 |
| | | | (0.015) | (0.028) |
| Declining | | | 0.155*** | 0.219 |
| | | | (0.024) | (0.043) |
| **Occ. Dest. Projected Outlook Categories (ref: Stable)** | | | | |
| Growing | | | 0.027† | 0.152*** |
| | | | (0.015) | (0.028) |
| Declining | | | -0.157*** | -0.009 |
| | | | (0.025) | (0.043) |
| **Occ. Origin** $t$ *** Occ. Dest.** $t+1$ | | | | |
| Growing * Growing | | | | 0.205*** |
| | | | | (0.035) |
| Growing * Declining | | | | -0.196*** |
| | | | | (0.055) |
| Declining * Growing | | | | -0.039 |
| | | | | (0.054) |
| Declining * Declining | | | | -0.242*** |
| | | | | (0.072) |

...continued

| | Whether a Mover Experienced Upward Mobility (5% or More Increase in Occupation-Level Earnings) | | | |
|---|---|---|---|---|
| | (1) | (2) | (3) | (4) |
| **Workers' Characteristics** | Yes | Yes | Yes | Yes |
| **Year Dummies** | Yes | Yes | Yes | Yes |
| **Intercept** | -0.299*** | -0.322*** | -0.246*** | -0.350*** |
| | (0.032) | (0.033) | (0.036) | (0.039) |
| **Number of Observations** | 96,977 | 96,977 | 96,977 | 96,977 |

*Notes*: Upward mobility is defined by whether the destination occupation's median earnings are at least 5% higher than the origin occupation's median earnings. The occupational growth rates over the last two years and over the next decades are quantified by increments of 10 percent. All models include workers' characteristics and year dummies as controls. Workers' characteristics include age, gender, race, ethnicity, and education.
$^{\dagger}p < 0.1$; $^{*}p < 0.05$; $^{**}p < 0.01$; $^{***}p < 0.001$; two-tailed tests.

# Part F: Auxiliary Analyses

The section includes supplementary tables and figures mentioned in the main paper.

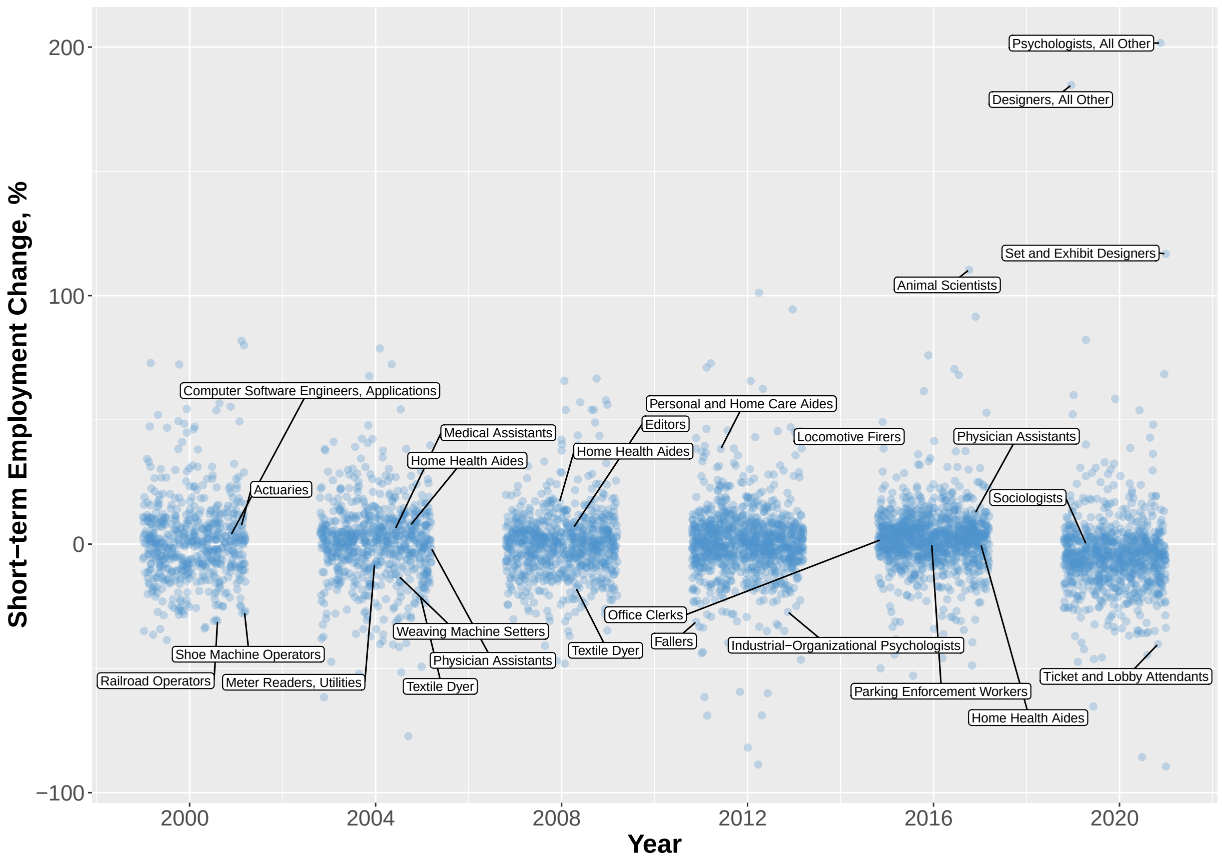


**Figure S5.** The Distribution of Occupations by 2-Year Short-term Occupational Growth Rates and Year

*Notes*: The figure shows occupational growth rates over the last two years between 2000 and 2020. For comparison purposes, we highlight the same occupations featured in Figure 1 as well as several fast-growing occupations with growth rates greater than 110%. Data from (*42–52*).

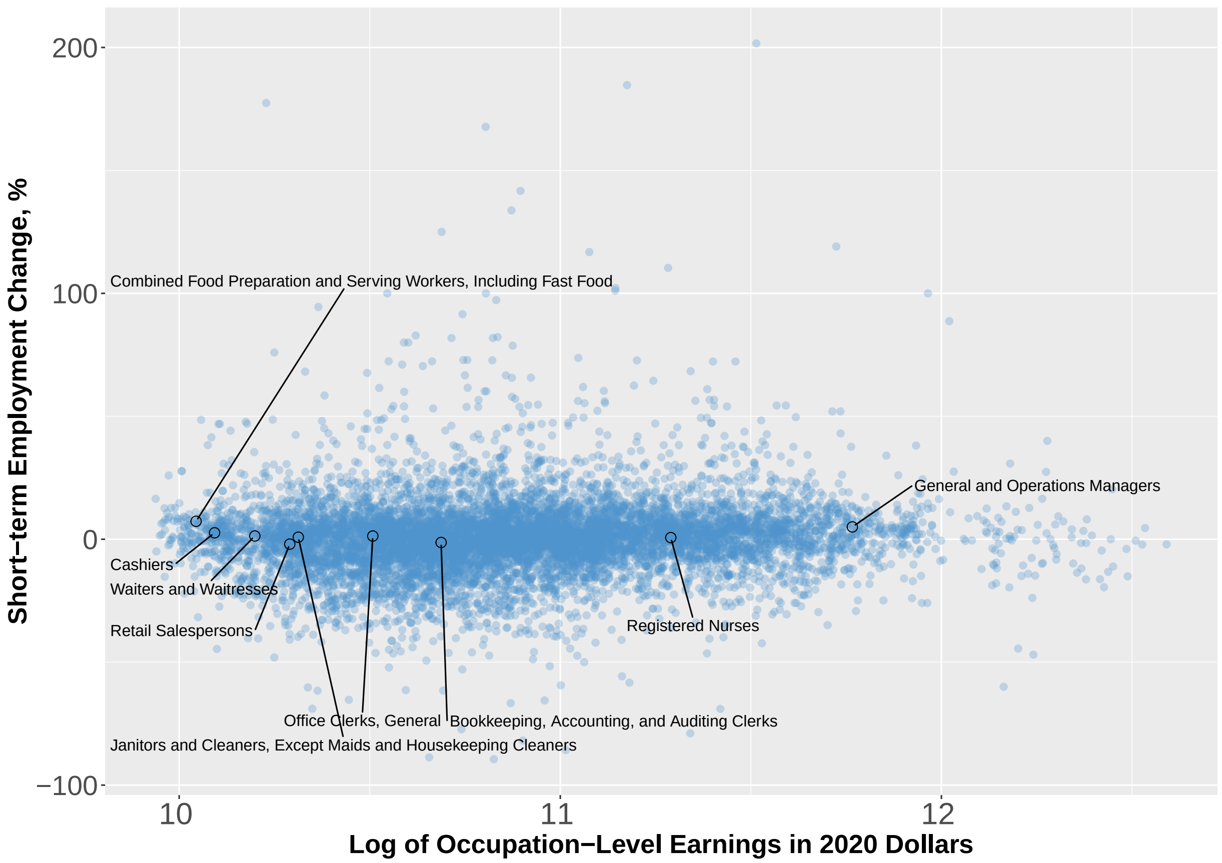


**Figure S6.** The Distribution of Occupations by 2-Year Short-term Occupational Growth Rates and Occupation-Level Annual Earnings

*Notes*: The figure shows occupational growth rates over the last two years across levels of annual earnings and highlights the top 15 largest occupations in terms of employment size. Data from OOH 2000–2020 are combined in this plot. For comparison purposes, we highlight the same occupations featured in Figure 1 of the main text.

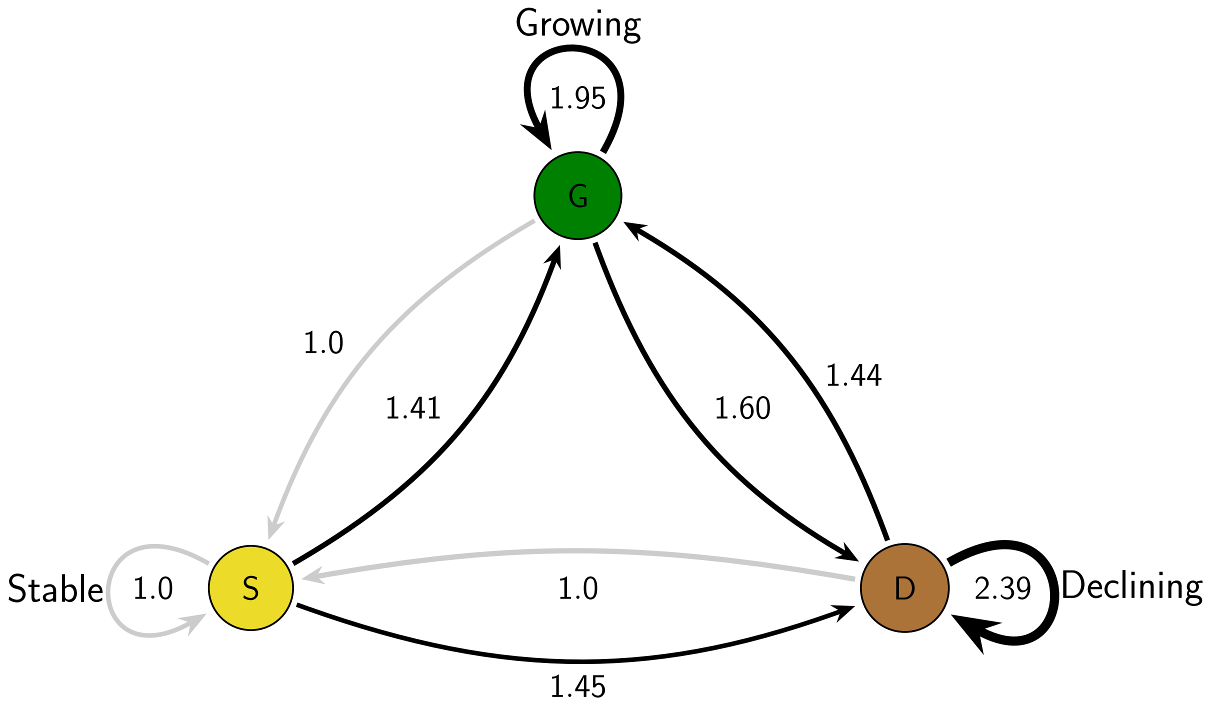


**Figure S7.** Odds Ratios from Discrete Choice Models Predicting Occupational Transitions Between Different Types of Short-Term Occupational Growth Categories

*Notes*: This plot displays odds ratios derived from the model estimates presented in *SM* Table S11, Model 2. The reference group consists of individuals who transition into stable occupations (indicated by grey lines). This serves as a relative baseline, with all other odds ratios scaled in comparison to it. We defined the short-term growth measure of occupations as growing or declining based on a $\pm 10\%$ change in employment over two years.

**Table S10.** Coefficients from Logistic Regression Models Predicting Occupational Changes Using Occupation-Level and Workers' Characteristics

| | Whether a Worker Changed Occupations Between Last and Current Years | |
|---|---|---|
| | (1) | (2) |
| **Occupational Growth Rate Over the Last Two Years (ref: Stable)** | | |
| Growing | 0.151*** | |
| | (0.011) | |
| Declining | 0.099*** | |
| | (0.012) | |
| **Projected Occupational Outlook Categories (ref: Slower Than Average)** | | |
| Much Faster Than Average | | -0.142*** |
| | | (0.016) |
| Faster Than Average | | 0.088*** |
| | | (0.015) |
| As Fast as Average | | -0.007 |
| | | (0.013) |
| Little or No Change | | -0.187*** |
| | | (0.015) |
| Decline | | 0.067*** |
| | | (0.017) |
| Decline Slowly or Moderately | | -0.094** |
| | | (0.029) |
| Decline Rapidly | | 0.005 |
| | | (0.044) |
| **Workers' Characteristics** | Yes | Yes |
| **Year Dummies** | Yes | Yes |
| **Number of Observations** | 921,829 | 921,829 |

*Notes*: Short-term occupational growth categories in Models (1) and (2) are defined by whether the number of jobs in an occupation has grown (or shrunk) by more than 10%, over the last two years. For long-term change, the eight categories of projected occupational outlook are defined in *SM* Table S 1. All models include workers' characteristics and year dummies as controls. Workers' characteristics include age, gender, race, ethnicity, and education.

$^{*}p < 0.05$; $^{**}p < 0.01$; $^{***}p < 0.001$; two-tailed tests.

**Table S11.** Coefficients from Discrete Choice Models Predicting Occupational Destinations Using Occupation-Level and Workers' Characteristics

| | Whether a Worker Moved into an Occupation in the Choice Set | | | |
|---|---|---|---|---|
| | (1) | (2) | (3) | (4) |
| **Occ. Dest. Growth Rate Over the Last Two Years (ref: Stable)** | | | | |
| Growing | 0.379*** | 0.345*** | | |
| | (0.064) | (0.065) | | |
| Declining | 0.544*** | 0.375*** | | |
| | (0.046) | (0.047) | | |
| **Occ. Origin Growth $t$ * Occ. Dest. Growth $t+1$ Over the Last Two Years** | | | | |
| Growing * Growing | | 0.321*** | | |
| | | (0.029) | | |
| Growing * Declining | | 0.092* | | |
| | | (0.041) | | |
| Declining * Growing | | 0.022 | | |
| | | (0.042) | | |
| Declining * Declining | | 0.496*** | | |
| | | (0.030) | | |
| **Occ. Dest. Projected Outlook Category (ref: Slower Than Average)** | | | | |
| Much Faster Than Average | | | -0.086 | -0.681*** |
| | | | (0.065) | (0.075) |
| Faster Than Average | | | -0.434*** | -0.651*** |
| | | | (0.059) | (0.065) |
| As Fast as Average | | | -0.232*** | -0.479*** |
| | | | (0.048) | (0.052) |
| Little or No Change | | | 0.069 | -0.072 |
| | | | (0.064) | (0.071) |
| Decline | | | -0.010 | -0.068 |
| | | | (0.064) | (0.071) |
| Decline Slowly or Moderately | | | 0.587*** | 0.345** |
| | | | (0.111) | (0.126) |
| Decline Rapidly | | | 0.623*** | 0.265 |

. . . continued

| | Whether a Worker Moved into an Occupation in the Choice Set | | | |
|---|---|---|---|---|
| | (1) | (2) | (3) | (4) |
| | | | (0.150) | (0.172) |
| **Occ. Origin Projected $t$ * Occ. Dest. Projected $t+1$** | | | | |
| Much Faster Than Average * Much Faster Than Average | | | | 1.908*** |
| | | | | (0.055) |
| Much Faster Than Average * Faster Than Average | | | | 0.784*** |
| | | | | (0.049) |
| Much Faster Than Average * As Fast as Average | | | | 0.603*** |
| | | | | (0.044) |
| Much Faster Than Average * Little or No Change | | | | -0.035 |
| | | | | (0.065) |
| Much Faster Than Average * Decline | | | | -0.321*** |
| | | | | (0.073) |
| Much Faster Than Average * Decline Slowly or Moderately | | | | 0.092 |
| | | | | (0.127) |
| Much Faster Than Average * Decline Rapidly | | | | 0.170 |
| | | | | (0.180) |
| Faster Than Average * Much Faster Than Average | | | | 0.978*** |
| | | | | (0.049) |
| Faster Than Average * Faster Than Average | | | | 0.694*** |
| | | | | (0.038) |
| Faster Than Average * As Fast as Average | | | | 0.410*** |
| | | | | (0.032) |
| Faster Than Average * Little or No Change | | | | -0.051 |
| | | | | (0.051) |
| Faster Than Average * Decline | | | | -0.256*** |
| | | | | (0.052) |
| Faster Than Average * Decline Slowly or Moderately | | | | 0.267* |
| | | | | (0.105) |
| Faster Than Average * Decline Rapidly | | | | 0.153 |
| | | | | (0.155) |

. . . continued

| | Whether a Worker Moved into an Occupation in the Choice Set | | | |
|---|---|---|---|---|
| | (1) | (2) | (3) | (4) |
| As Fast as Average * Much Faster Than Average | | | | 0.569*** |
| | | | | (0.044) |
| As Fast as Average * Faster Than Average | | | | 0.308*** |
| | | | | (0.032) |
| As Fast as Average * As Fast as Average | | | | 0.434*** |
| | | | | (0.026) |
| As Fast as Average * Little or No Change | | | | 0.064 |
| | | | | (0.040) |
| As Fast as Average * Decline | | | | -0.179*** |
| | | | | (0.041) |
| As Fast as Average * Decline Slowly or Moderately | | | | 0.179* |
| | | | | (0.087) |
| As Fast as Average * Decline Rapidly | | | | 0.256* |
| | | | | (0.121) |
| Little or No Change * Much Faster Than Average | | | | 0.032 |
| | | | | (0.063) |
| Little or No Change * Faster Than Average | | | | -0.148* |
| | | | | (0.049) |
| Little or No Change * As Fast as Average | | | | 0.082* |
| | | | | (0.039) |
| Little or No Change * Little or No Change | | | | 0.323*** |
| | | | | (0.050) |
| Little or No Change * Decline | | | | 0.176*** |
| | | | | (0.054) |
| Little or No Change * Decline Slowly or Moderately | | | | 0.591*** |
| | | | | (0.108) |
| Little or No Change * Decline Rapidly | | | | 0.788*** |
| | | | | (0.143) |
| Decline * Much Faster Than Average | | | | -0.162* |
| | | | | (0.069) |

. . . continued

| | Whether a Worker Moved into an Occupation in the Choice Set | | | |
|---|---|---|---|---|
| | (1) | (2) | (3) | (4) |
| Decline * Faster Than Average | | | | -0.182*** |
| | | | | (0.051) |
| Decline * As Fast as Average | | | | -0.048 |
| | | | | (0.041) |
| Decline * Little or No Change | | | | 0.319*** |
| | | | | (0.055) |
| Decline * Decline | | | | 0.456*** |
| | | | | (0.049) |
| Decline * Decline Slowly or Moderately | | | | - |
| Decline * Decline Rapidly | | | | - |
| Decline Slowly or Moderately * Much Faster Than Average | | | | 0.264* |
| | | | | (0.122) |
| Decline Slowly or Moderately * Faster Than Average | | | | 0.117 |
| | | | | (0.103) |
| Decline Slowly or Moderately * As Fast as Average | | | | 0.069 |
| | | | | (0.083) |
| Decline Slowly or Moderately * Little or No Change | | | | 0.565*** |
| | | | | (0.102) |
| Decline Slowly or Moderately * Decline | | | | - |
| Decline Slowly or Moderately * Decline Slowly or Moderately | | | | 0.602*** |
| | | | | (0.128) |
| Decline Slowly or Moderately * Decline Rapidly | | | | 1.167*** |
| | | | | (0.155) |
| Decline Rapidly * Much Faster Than Average | | | | 0.428* |
| | | | | (0.197) |
| Decline Rapidly * Faster Than Average | | | | 0.389* |
| | | | | (0.162) |

…continued

| | Whether a Worker Moved into an Occupation in the Choice Set | | | |
|---|---|---|---|---|
| | (1) | (2) | (3) | (4) |
| Decline Rapidly * As Fast as Average | | | | 0.252† |
| | | | | (0.136) |
| Decline Rapidly * Little or No Change | | | | 0.767*** |
| | | | | (0.161) |
| Decline Rapidly * Decline | | | | - |
| Decline Rapidly * Decline Slowly or Moderately | | | | 1.122*** |
| | | | | (0.176) |
| Decline Rapidly * Decline Rapidly | | | | 1.865*** |
| | | | | (0.192) |
| **Workers' Characteristics** | Yes | Yes | Yes | Yes |
| **Year Dummies** | Yes | Yes | Yes | Yes |
| **Occupational Size Constaint** | Yes | Yes | Yes | Yes |
| **Number of Workers** | 104,457 | 104,457 | 104,457 | 104,457 |
| **Number of Observations** | 42,733,727 | 41,855,329 | 42,733,727 | 42,733,727 |

*Notes*: Short-term occupational growth categories in Models (1) and (2) are defined by whether the number of jobs in an occupation has grown (or shrunk) by more than 10%, over the last two years. For long-term occupational change, the eight categories of projected occupational outlook are defined in *SM* Table S1. All models control for workers' characteristics and year dummies, which are added as interactions between these variables and the characteristics of occupations in the choice set. Workers' characteristics include age, gender, race, ethnicity, and education. The interaction terms between some origin and destination occupational outlook categories are omitted due to zero observations in such combinations.
$^{\dagger}p < 0.1$; $^{*}p < 0.05$; $^{**}p < 0.01$; $^{***}p < 0.001$; two-tailed tests.

**Table S12.** Logistic Regression Coefficients Predicting Upward Mobility Using Occupation-Level and Workers' Characteristics

| | Whether a Mover Experienced Upward Mobility (5% or More Increase in Occupation-Level Earnings) | | | |
|---|---|---|---|---|
| | (1) | (2) | (3) | (4) |
| **Occ. Origin Growth Rate Over the Last Two Years (ref: Stable)** | | | | |
| Growing | 0.080*** | 0.156*** | | |
| | (0.020) | (0.023) | | |
| Declining | -0.211*** | -0.217*** | | |
| | (0.023) | (0.028) | | |
| **Occ. Dest. Growth Rate Over the Last Two Years (ref: Stable)** | | | | |
| Growing | 0.152*** | 0.172*** | | |
| | (0.021) | (0.023) | | |
| Declining | 0.205*** | 0.272*** | | |
| | (0.023) | (0.027) | | |
| **Occ. Origin Growth** $t$ *** Occ. Dest. Growth** $t+1$ | | | | |
| Growing * Growing | | -0.252*** | | |
| | | (0.056) | | |
| Growing * Declining | | -0.414*** | | |
| | | (0.075) | | |
| Declining * Growing | | 0.245** | | |
| | | (0.077) | | |
| Declining * Declining | | -0.106† | | |
| | | (0.056) | | |
| **Occ. Orig. Projected Outlook Category (ref: Slower Than Average)** | | | | |
| Much Faster Than Average | | | 0.309*** | 0.126† |
| | | | (0.027) | (0.074) |
| Faster Than Average | | | 0.073** | 0.108* |
| | | | (0.023) | (0.053) |
| As Fast as Average | | | -0.026 | 0.255*** |
| | | | (0.019) | (0.042) |

. . . continued

| | Whether a Mover Experienced Upward Mobility (5% or More Increase in Occupation-Level Earnings) | | | |
|---|---|---|---|---|
| | (1) | (2) | (3) | (4) |
| Little or No Change | | | 0.466*** | 0.342*** |
| | | | (0.027) | (0.061) |
| Decline | | | 0.266*** | 0.479*** |
| | | | (0.029) | (0.063) |
| Decline Slowly or Moderately | | | 0.558*** | 0.969*** |
| | | | (0.054) | (0.140) |
| Decline Rapidly | | | 0.500*** | 0.684** |
| | | | (0.082) | (0.227) |
| **Occ. Dest. Projected Outlook Category (ref: Slower Than Average)** | | | | |
| Much Faster Than Average | | | -0.022 | 0.005 |
| | | | (0.027) | (0.074) |
| Faster Than Average | | | 0.082*** | 0.202*** |
| | | | (0.023) | (0.052) |
| As Fast as Average | | | -0.203*** | -0.021 |
| | | | (0.019) | (0.042) |
| Little or No Change | | | -0.385*** | -0.205** |
| | | | (0.028) | (0.063) |
| Decline | | | -0.259*** | -0.250*** |
| | | | (0.029) | (0.064) |
| Decline Slowly or Moderately | | | -0.399*** | -0.156 |
| | | | (0.057) | (0.146) |
| Decline Rapidly | | | -0.383*** | 0.417* |
| | | | (0.077) | (0.196) |
| **Occ. Origin Projected** $t$ *** Occ. Dest. Projected** $t+1$ | | | | |
| Much Faster Than Average * Much Faster Than Average | | | | 0.184† |
| | | | | (0.107) |
| Much Faster Than Average * Faster Than Average | | | | -0.119 |
| | | | | (0.097) |
| Much Faster Than Average * As Fast as Average | | | | 0.448*** |

. . . continued

| | Whether a Mover Experienced Upward Mobility (5% or More Increase in Occupation-Level Earnings) | | | |
|---|---|---|---|---|
| | (1) | (2) | (3) | (4) |
| | | | | (0.086) |
| Much Faster Than Average * Little or No Change | | | | 0.308* |
| | | | | (0.128) |
| Much Faster Than Average * Decline | | | | -0.067 |
| | | | | (0.144) |
| Much Faster Than Average * Decline Slowly or Moderately | | | | -0.430 |
| | | | | (0.264) |
| Much Faster Than Average * Decline Rapidly | | | | -0.647† |
| | | | | (0.359) |
| Faster Than Average * Much Faster Than Average | | | | 0.131 |
| | | | | (0.097) |
| Faster Than Average * Faster Than Average | | | | -0.110 |
| | | | | (0.075) |
| Faster Than Average * As Fast as Average | | | | -0.055 |
| | | | | (0.065) |
| Faster Than Average * Little or No Change | | | | -0.066 |
| | | | | (0.101) |
| Faster Than Average * Decline | | | | 0.048 |
| | | | | (0.105) |
| Faster Than Average * Decline Slowly or Moderately | | | | -0.079 |
| | | | | (0.212) |
| Faster Than Average * Decline Rapidly | | | | -1.053** |
| | | | | (0.323) |
| As Fast as Average * Much Faster Than Average | | | | -0.178* |
| | | | | (0.087) |
| As Fast as Average * Faster Than Average | | | | -0.141* |
| | | | | (0.064) |
| As Fast as Average * As Fast as Average | | | | -0.504*** |
| | | | | (0.052) |

. . . continued

| | Whether a Mover Experienced Upward Mobility (5% or More Increase in Occupation-Level Earnings) | | | |
|---|---|---|---|---|
| | (1) | (2) | (3) | (4) |
| As Fast as Average * Little or No Change | | | | -0.305*** |
| | | | | (0.080) |
| As Fast as Average * Decline | | | | 0.008 |
| | | | | (0.082) |
| As Fast as Average * Decline Slowly or Moderately | | | | -0.462** |
| | | | | (0.173) |
| As Fast as Average * Decline Rapidly | | | | -0.846*** |
| | | | | (0.238) |
| Little or No Change * Much Faster Than Average | | | | 0.102 |
| | | | | (0.124) |
| Little or No Change * Faster Than Average | | | | 0.084 |
| | | | | (0.096) |
| Little or No Change * As Fast as Average | | | | 0.215** |
| | | | | (0.076) |
| Little or No Change * Little or No Change | | | | -0.014 |
| | | | | (0.099) |
| Little or No Change * Decline | | | | 0.277** |
| | | | | (0.103) |
| Little or No Change * Decline Slowly or Moderately | | | | 0.271 |
| | | | | (0.211) |
| Little or No Change * Decline Rapidly | | | | -0.568* |
| | | | | (0.278) |
| Decline * Much Faster Than Average | | | | 0.163 |
| | | | | (0.136) |
| Decline * Faster Than Average | | | | -0.227* |
| | | | | (0.100) |
| Decline * As Fast as Average | | | | -0.268*** |
| | | | | (0.080) |
| Decline * Little or No Change | | | | -0.444*** |

. . . continued

| | Whether a Mover Experienced Upward Mobility (5% or More Increase in Occupation-Level Earnings) | | | |
|---|---|---|---|---|
| | (1) | (2) | (3) | (4) |
| | | | | (0.106) |
| Decline * Decline | | | | -0.214* |
| | | | | (0.097) |
| Decline * Decline Slowly or Moderately | | | | - |
| Decline * Decline Rapidly | | | | - |
| Decline Slowly or Moderately * Much Faster Than Average | | | | -0.129 |
| | | | | (0.243) |
| Decline Slowly or Moderately * Faster Than Average | | | | -0.721*** |
| | | | | (0.203) |
| Decline Slowly or Moderately * As Fast as Average | | | | -0.264 |
| | | | | (0.164) |
| Decline Slowly or Moderately * Little or No Change | | | | -0.735*** |
| | | | | (0.201) |
| Decline Slowly or Moderately * Decline | | | | - |
| Decline Slowly or Moderately * Decline Slowly or Moderately | | | | -0.849*** |
| | | | | (0.253) |
| Decline Slowly or Moderately * Decline Rapidly | | | | -1.458*** |
| | | | | (0.307) |
| Decline Rapidly * Much Faster Than Average | | | | 0.288 |
| | | | | (0.400) |
| Decline Rapidly * Faster Than Average | | | | -0.468 |
| | | | | (0.322) |
| Decline Rapidly * As Fast as Average | | | | -0.189 |
| | | | | (0.269) |
| Decline Rapidly * Little or No Change | | | | -0.534 |
| | | | | (0.323) |

…continued

| | Whether a Mover Experienced Upward Mobility (5% or More Increase in Occupation-Level Earnings) | | | |
|---|---|---|---|---|
| | (1) | (2) | (3) | (4) |
| Decline Rapidly * Decline | | | | - |
| Decline Rapidly * Decline Slowly or Moderately | | | | 0.177 |
| | | | | (0.348) |
| Decline Rapidly * Decline Rapidly | | | | -1.237** |
| | | | | (0.385) |
| **Workers' Characteristics** | Yes | Yes | Yes | Yes |
| **Year Dummies** | Yes | Yes | Yes | Yes |
| **Intercept** | -0.206*** | -0.213*** | -0.303*** | -0.413*** |
| | (0.034) | (0.034) | (0.039) | (0.046) |
| **Number of Observations** | 96,977 | 96,977 | 96,977 | 96,977 |

*Notes*: Short-term occupational growth categories in Models (1) and (2) are defined by whether the number of jobs in an occupation has grown (or shrunk) by more than 10%, over the last two years. For long-term occupational change, the eight categories of projected occupational outlook are defined in *SM* Table S1. All models control for workers' characteristics and year dummies, which are added as interactions between these variables and the characteristics of occupations in the choice set. Workers' characteristics include age, gender, race, ethnicity, and education. The interaction terms between some origin and destination occupational outlook categories are omitted due to zero observations in such combinations.
$^{\dagger}p < 0.1$; $^{*}p < 0.05$; $^{**}p < 0.01$; $^{***}p < 0.001$; two-tailed tests.

**Table S13.** The Distributions of Vertical Mobility Among Movers

| Vertical Mobility | Definitions Based on<br>5% Change in<br>Occupation-Level Earnings | <br>$1,000 Change in<br>Occupation-Level Earnings |
|---|---|---|
| Upward | 38.64 | 46.74 |
| Downward | 39.26 | 47.72 |
| Horizontal | 22.10 | 5.54 |
| All | 100.00 | 100.00 |

*Notes*: We define upward mobility by (1) whether the destination occupation's median earnings are at least 5% higher than the origin occupation's median earnings or (2) whether the destination occupation's median earnings are at least $1,000 higher than the origin occupation's median earnings.

**Table S14.** Coefficients from Logistic Regression Models Predicting Upward Mobility Using Occupation-Level and Workers' Characteristics

| | Whether a Mover Experienced Upward Mobility (At Least $1,000 Increase in Occupation-Level Earnings) | | | |
|---|---|---|---|---|
| | (1) | (2) | (3) | (4) |
| **Occ. Origin Growth Rate Over the Last Two Years** | -0.0006*** | | | |
| | (0.0002) | | | |
| **Occ. Dest. Growth Rate Over the Last Two Years** | 0.0008*** | | | |
| | (0.0002) | | | |
| **Occ. Origin Projected Growth Rate Over the Next Decade** | | -0.009 | | |
| | | (0.007) | | |
| **Occ. Dest. Projected Growth Rate Over the Next Decade** | | 0.029*** | | |
| | | (0.007) | | |
| **Occ. Origin Projected Outlook Categories (ref: Stable)** | | | | |
| Growing | | | -0.100*** | 0.038 |
| | | | (0.015) | (0.028) |
| Declining | | | 0.173*** | 0.233*** |
| | | | (0.024) | (0.043) |
| **Occ. Dest. Projected Outlook Categories (ref: Stable)** | | | | |
| Growing | | | 0.016 | 0.136*** |
| | | | (0.015) | (0.028) |
| Declining | | | -0.169*** | -0.039 |
| | | | (0.025) | (0.043) |
| **Occ. Origin** $t$ *** Occ. Dest.** $t+1$ | | | | |
| Growing * Growing | | | | -0.194*** |
| | | | | (0.034) |
| Growing * Declining | | | | -0.173** |
| | | | | (0.055) |
| Declining * Growing | | | | -0.042 |
| | | | | (0.054) |
| Declining * Declining | | | | -0.207** |
| | | | | (0.072) |

. . . continued

| | Whether a Mover Experienced Upward Mobility (At Least $1,000 Increase in Occupation-Level Earnings) | | | |
|---|---|---|---|---|
| | (1) | (2) | (3) | (4) |
| **Workers' Characteristics** | Yes | Yes | Yes | Yes |
| **Year Dummies** | Yes | Yes | Yes | Yes |
| **Intercept** | -0.183*** | -0.196*** | -0.144*** | -0.226*** |
| | (0.032) | (0.032) | (0.035) | (0.039) |
| **Number of Observations** | 96,977 | 96,977 | 96,977 | 96,977 |

*Notes*: Upward mobility is defined by whether the destination occupation's median earnings are $1,000 higher than the origin occupation's median earnings. The occupational growth rates over the last two years and over the next decades are quantified by increments of 10 percent. All models include workers' characteristics and year dummies as controls. Workers' characteristics include age, gender, race, ethnicity, and education.

$^{*}p < 0.05$; $^{**}p < 0.01$; $^{***}p < 0.001$; two-tailed tests.

**Table S15.** Types of Mobility by Occupational Origins

| | Mobility into . . . | | | | | |
|---|---|---|---|---|---|---|
| Origin Occupation | Growing | Stable | Declining | Unemployed | Out of the Labor Force | Total |
| Growing | 40,861 | 12,028 | 4,035 | 4,894 | 630 | 62,448 |
| Stable | 12,834 | 7,462 | 2,854 | 2,206 | 228 | 25,584 |
| Declining | 4,452 | 2,765 | 1,964 | 768 | 92 | 10,041 |
| All | 58,147 | 22,255 | 8,853 | 7,868 | 950 | 98,073 |
| | Growing | Stable | Declining | Unemployed | Out of the Labor Force | Total |
| Growing | 65.43 | 19.26 | 6.46 | 7.84 | 1.01 | 100.00 |
| Stable | 50.16 | 29.17 | 11.16 | 8.62 | 0.89 | 100.00 |
| Declining | 44.34 | 27.54 | 19.56 | 7.65 | 0.92 | 100.00 |

*Notes*: Occupational origins refer to projected 10-year outlook categories.

**Table S16.** Odds Ratios from Discrete Choice Models Predicting Occupational Transitions Between Different Types of Occupational Growth Categories

| **Panel A: Stable Occupations as the Reference Group** | | |
|---|---|---|
| Origin → Destination | Odds Ratio | |
| **Panel C: Declining Occupations as the Reference Group** | | |
| Origin → Destination | Relative Likelihood | Calculation |
| Stable → Declining | 1.00 | Reference |
| Stable → Stable | 0.99 | 1.00/1.01 |
| Stable → Growing | 0.59 | 0.60/1.01 |
| Growing → Stable | 1.20 | 1.00/0.83 |
| Growing → Growing | 1.27 | 1.05/0.83 |
| Growing → Declining | 1.00 | Reference |
| Declining → Stable | 0.68 | 1.00/1.46 |
| Declining → Growing | 0.36 | 0.52/1.46 |
| Declining → Declining | 1.00 | Reference |

*Notes*: Panel A shows odds ratios calculated from Model 4 results in Table S8 and presented in Figure 4. The reference group consists of individuals who transition into stable occupations. This serves as a relative baseline, with all other odds ratios scaled in comparison to it. Values equal to 1.00 indicate transitions with identical likelihood to the reference, values greater than 1.00 show higher likelihood, and values less than 1.00 demonstrate lower likelihood relative to the chosen baseline transition. By definition, the odds of transitioning from a growing or declining origin into a stable destination serve as the reference within each origin group and are set to 1.0. Panel B and Panel C re-express the same transition odds using growing and declining occupations as alternative reference groups, respectively. The calculation method preserves underlying probability relationships by dividing any two odds ratios from the same origin group from Panel A.

**Table S17.** Coefficients from Logistic Regression Models Predicting Upward Mobility Using Individual Level Earnings Changes Based on 1-Year CPS-ASEC Linked Panel

| | Whether a Mover Experienced Upward Mobility (5% or More Increase in Occupation-Level Earnings) | | | |
|---|---|---|---|---|
| | (1) | (2) | (3) | (4) |
| **Occ. Origin Growth Rate Over the Last Two Years** | -0.00004 | | | |
| | (0.0005) | | | |
| **Occ. Dest. Growth Rate Over the Last Two Years** | -0.00005 | | | |
| | (0.0006) | | | |
| **Occ. Origin Projected Growth Rate Over the Next Decade** | | 0.059*** | | |
| | | (0.016) | | |
| **Occ. Dest. Projected Growth Rate Over the Next Decade** | | -0.007 | | |
| | | (0.016) | | |
| **Occ. Origin Projected Outlook Categories (ref: Stable)** | | | | |
| Growing | | | 0.082* | -0.065 |
| | | | (0.036) | (0.067) |
| Declining | | | -0.037 | -0.108 |
| | | | (0.057) | (0.103) |
| **Occ. Dest. Projected Outlook Categories (ref: Stable)** | | | | |
| Growing | | | -0.002 | -0.133* |
| | | | (0.036) | (0.067) |
| Declining | | | -0.018 | -0.166 |
| | | | (0.058) | (0.101) |
| **Occ. Origin** $t$ *** Occ. Dest.** $t+1$ | | | | |
| Growing * Growing | | | | 0.202* |
| | | | | (0.081) |
| Growing * Declining | | | | 0.256$^{\dagger}$ |
| | | | | (0.131) |
| Declining * Growing | | | | 0.108 |
| | | | | (0.130) |
| Declining * Declining | | | | 0.117 |
| | | | | (0.168) |

. . . continued

| | Whether a Mover Experienced Upward Mobility (5% or More Increase in Occupation-Level Earnings) | | | |
|---|---|---|---|---|
| | (1) | (2) | (3) | (4) |
| **Workers' Characteristics** | Yes | Yes | Yes | Yes |
| **Year Dummies** | Yes | Yes | Yes | Yes |
| **Intercept** | 0.032 | 0.008 | 0.004 | 0.091 |
| | (0.076) | (0.077) | (0.084) | (0.090) |
| **Number of Observations** | 17,362 | 17,362 | 17,362 | 17,362 |

*Notes*: Upward mobility is defined as a worker's current earnings being at least 5% higher than their earnings in the previous year, among those who changed occupations between the two years. The analyses use the linked 1-year CPS panel to measure individual earnings changes rather than changes in occupation-level earnings. However, the sample size for this analysis is quite small. Each year includes only about 1,000 worker observations, and at the occupational level, there are likely just a few cases per group. Most of the coefficients in the resulting table are not statistically significant.
$^{\dagger}p < 0.1$; $^{*}p < 0.05$; $^{**}p < 0.01$; $^{***}p < 0.001$; two-tailed tests.

**Table S18.** Discrete Choice Model Coefficients Predicting Occupational Destinations Using Projected Job Vacancies in the Next Ten Years as a Model Constraint

| | Whether a Worker Moved into an Occupation in the Choice Set | | | |
|---|---|---|---|---|
| | (1) | (2) | (3) | (4) |
| **Occ. Dest. Growth Rate Over the Last Two Years** | -0.114*** | | | |
| | (0.012) | | | |
| **Occ. Dest. Projected Growth Over the Next Decade** | | -0.222*** | | |
| | | (0.016) | | |
| **Occ. Dest. Projected Outlook Category (ref: Stable)** | | | | |
| Growing | | | -0.376*** | -0.646*** |
| | | | (0.037) | (0.040) |
| Declining | | | 0.102 | 0.079 |
| | | | (0.055) | (0.058) |
| **Occ. Origin** $t$ *** Occ. Dest.** $t+1$ | | | | |
| Growing * Growing | | | | 0.558*** |
| | | | | (0.017) |
| Growing * Declining | | | | -0.203*** |
| | | | | (0.028) |
| Declining * Growing | | | | -0.152*** |
| | | | | (0.028) |
| Declining * Declining | | | | 0.367*** |
| | | | | (0.036) |
| **Workers' Characteristics** | Yes | Yes | Yes | Yes |
| **Year Dummies** | Yes | Yes | Yes | Yes |
| **Occupational Size Constaint** | Yes | Yes | Yes | Yes |
| **Number of Workers** | 104,457 | 104,457 | 104,457 | 104,457 |
| **Number of Observations** | 42,196,783 | 42,196,783 | 42,196,783 | 42,196,783 |

*Notes*: The discrete choice models are presented in equations (7)–(9). They follow the same structure as those in *SM* Table S8, with one key difference: instead of using employment size as the model constraint $\mathbf{D}_{kj}$, we use BLS projections of job vacancies for each occupation over the next ten years. These projections are available from the BLS occupation-industry matrix, which reports annual average occupational openings for 2023–2033 (see Table 1.10, column N, `https://www.bls.gov/emp/tables/industry-occupation-matrix-industry.htm`, accessed January 8, 2026). For earlier years, comparable tables appear in printed BLS publications, though they are not available online. We have digitized these historical

tables, and the data are available upon request.
$^{*}p < 0.05$; $^{**}p < 0.01$; $^{***}p < 0.001$; two-tailed tests.

## REFERENCES


1. W. J. Wilson, *When Work Disappears: The World of the New Urban Poor* (Alfred A. Knopf, 1996).

2. D. H. Autor, D. Dorn, G. H. Hanson, The China shock: Learning from labor-market adjustment to large changes in trade. *Annu. Rev. Econ.* **8**, 205–240 (2016).

3. D. Autor, C. Chin, A. Salomons, B. Seegmiller, New frontiers: The origins and content of New Work, 1940–2018. *Q. J. Econ.* **139**, 1399–1465 (2024).

4. J. E. Brand, The far-reaching impact of job loss and unemployment. *Annu. Rev. Sociol.* **41**, 359–375 (2015).

5. E. Brynjolfsson, A. McAfee, *The Second Machine Age: Work, Progress, and Prosperity in a Time of Brilliant Technologies* (WW Norton & Company, 2014).

6. R. E. Dwyer, The care economy? Gender, economic restructuring, and job polarization in the U.S. labor market. *Am. Sociol. Rev.* **78**, 390–416 (2013).

7. B. Jovanovic, R. Moffitt, An estimate of a sectoral model of labor mobility. *J. Polit. Econ.* **98**, 827–852 (1990).

8. M. Kuhn, I. Manovskii, X. Qiu, "The geography of job creation and job destruction" (Tech. Rep. NBER working paper no. 29399, National Bureau of Economic Research (2021).

9. M. J. Handel, "Growth trends for selected occupations considered at risk from automation," in *Monthly Labor Review* (U.S. Bureau of Labor Statistics, 2022); https://doi.org/10.21916/mlr.2022.21.

10. M. Lee, M. Mather, *Population Bulletin US Labor Force Trends* (Population Reference Bureau, 2008), vol. 63.

11. A. Ollier-Malaterre, J. A. Jacobs, N. P. Rothbard, Technology, work, and family: Digital cultural capital and boundary management. *Annu. Rev. Sociol.* **45**, 425–447 (2019).

12. B. Shestakofsky, Working algorithms: Software automation and the future of work. *Work Occupat.* **44**, 376–423 (2017).

13. M. L. Dolfman, M. Insco, R. J. Holden, "Healthcare jobs and the great recession," in *Monthly Labor Review* (U.S. Bureau of Labor Statistics, 2018); https://doi.org/10.21916/mlr.2018.17.

14. A. Hogan, B. Roberts, "Occupational employment projections to 2024," in *Monthly Labor Review* (U.S. Bureau of Labor Statistics, 2015); https://doi.org/10.21916/mlr.2015.49.

15. O. D. Duncan, "Methodological issues in the analysis of social mobility," in *Social Structure and Mobility in Economic Development*, N. J. Smelser, S. M. Lipset, Eds. (Aldine, 1996), pp. 51–97.

16. M. E. Sobel, Structural mobility, circulation mobility and the analysis of occupational mobility: A conceptual mismatch. *Am. Sociol. Rev.* **48**, 721–727 (1983).

17. M. E. Sobel, M. Hout, O. D. Duncan, Exchange, structure, and symmetry in occupational mobility. *Am. J. Sociol.* **91**, 359–372 (1985).

18. Y. Xie, A. Killewald, Intergenerational occupational mobility in Great Britain and the United States since 1850: Comment. *Am. Econ. Rev.* **103**, 2003–2020 (2013).

19. M. Hout, More universalism, less structural mobility: The American occupational structure in the 1980s. *Am. J. Sociol.* **93**, 1358–1400 (1988).

20. R. M. Hauser, P. J. Dickinson, H. P. Travis, J. N. Koffel, Structural changes in occupational mobility among men in the United States. *Am. Sociol. Rev.* **40**, 585–598 (1975).

21. R. Erikson, J. H. Goldthorpe, *The Constant Flux: A Study of Class Mobility in Industrial Societies* (Oxford Univ. Press, 1992).

22. J. O. Jonsson, D. B. Grusky, M. Di Carlo, R. Pollak, M. C. Brinton, Microclass mobility: Social reproduction in four countries. *Am. J. Sociol.* **114**, 977–1036 (2009).

23. J. B. Sørensen, D. B. Grusky, “The Structure of career mobility in microscopic perspective,” in *Social Differentiation and Social Inequality*, J. N. Baron, D. B. Grusky, D. J. Treiman, Eds. (Routledge, 1996), pp. 83–114.

24. K. A. Weeden, D. B. Grusky, The case for a new class map. *Am. J. Sociol.* **111**, 141–212 (2005).

25. K. A. Weeden, D. B. Grusky, The three worlds of inequality. *Am. J. Sociol.* **117**, 1723–1785 (2012).

26. T. A. DiPrete, K. L. Nonnemaker, Structural change, labor market turbulence, and labor market outcomes. *Am. Sociol. Rev.* **62**, 386–404 (1997).

27. T. A. DiPrete, Industrial restructuring and the mobility response of American workers in the 1980s. *Am. Sociol. Rev.* **58**, 74–96 (1993).

28. K. B. Karlson, B. Jann, Marginal odds ratios: What they are, how to compute them, and why sociologists might want to use them. *Sociol. Sci.* **10**, 332–347 (2023).

29. C. Mood, Logistic regression: Why we cannot do what we think we can do, and what we can do about it. *Eur. Sociol. Rev.* **26**, 67–82 (2010).

30. T. Mouw, A. L. Kalleberg, Stepping stone versus dead end jobs: Occupational pathways out of working poverty in the United States, 1996–2012. *Am. Sociol. Rev.* **89**, 298–345 (2024).

31. W. J. Carrington, B. Fallick, Why do earnings fall with job displacement? *Ind. Relat.* **56**, 688–722 (2017).

32. B. Fallick, J. Haltiwanger, E. McEntarfer, M. Staiger, Job displacement and earnings losses: The role of joblessness. *Am. Econ. J. Macroecon.* **17**, 177–205 (2025).

33. L. S. Jacobson, R. J. LaLonde, D. G. Sullivan, Earnings losses of displaced workers. *Am. Econ. Rev.* , 685–709 (1993).

34. K. A. Couch, D. W. Placzek, Earnings losses of displaced workers revisited. *Am. Econ. Rev.* **100**, 572–589 (2010).

35. D. J. Deming, The growing importance of social skills in the labor market. *Q. J. Econ.* **132**, 1593–1640 (2017).

36. A. Spitz-Oener, Technical change, job tasks, and rising educational demands: Looking outside the wage structure. *J. Labor Econ.* **24**, 235–270 (2006).

37. H. Goldstein, "The early history of the occupational outlook handbook," in *Monthly Labor Review* (U.S. Bureau of Labor Statistic, 1999); www.bls.gov/opub/mlr/1999/05/art1full.pdf.

38. T. L. Morisi, "70 years of the occupational outlook handbook," in *Monthly Labor Review* (U.S. Bureau of Labor Statistics, 2019); https://doi.org/10.21916/mlr.2019.28.

39. M. J. Pilot, "Occupational outlook handbook: A review of 50 years of change," in *Monthly Labor Review* (U.S. Bureau of Labor Statistic, 1999); www.bls.gov/opub/mlr/1999/05/art2full.pdf.

40. S. Flood, M. King, R. Rodgers, S. Ruggles, J. Robert Warren, M. Westberry, Integrated Public Use Microdata Series, Current Population Survey: Version 9.0, IPUMS (2021); https://doi.org/10.18128/D030.V9.0.

41. A. Gayfield, L. Laughlin, "Counting the Hustle: Platform workers and digital entrepreneurship in federal household surveys," Working paper, Census Bureau, U.S. Census Bureau, Washington, DC, 2023.

42. U.S. Department of Labor, "Occupational Outlook Handbook "(U.S. Government Printing Office, ed. 2000–01, Washington, DC, 2000).

43. U.S. Department of Labor, "Occupational Outlook Handbook" (U.S. Government Printing Office, ed. 2002–03, Washington, DC, 2002).

44. U.S. Department of Labor, "Occupational Outlook Handbook" (U.S. Government Printing Office, ed. 2004–05, Washington, DC, 2004).

45. U.S. Department of Labor, “Occupational Outlook Handbook” (U.S. Government Printing Office, ed. 2006–07, Washington, DC, 2006).

46. U.S. Department of Labor, “Occupational Outlook Handbook” (U.S. Government Printing Office, ed. 2008–09, Washington, DC, 2008).

47. U.S. Department of Labor, “Occupational Outlook Handbook” (U.S. Government Printing Office, ed. 2010–11, Washington, DC, 2010).

48. U.S. Department of Labor, “Occupational Outlook Handbook” (U.S. Government Printing Office, ed. 2012–13, Washington, DC, 2012).

49. U.S. Department of Labor, “Occupational Outlook Handbook” (U.S. Government Printing Office, ed. 2014–15, Washington, DC, 2014).

50. U.S. Department of Labor, “Occupational Outlook Handbook” (U.S. Government Printing Office, ed. 2016–17, Washington, DC, 2016).

51. U.S. Department of Labor, “Occupational Outlook Handbook” (U.S. Government Printing Office, ed. 2018–19, Washington, DC, 2018).

52. U.S. Department of Labor, “Occupational Outlook Handbook” (U.S. Government Printing Office, ed. 2020–21, Washington, DC, 2019).

53. J. Levine, J. Nottingham, B. Paige, P. Lewis, “Transitioning O*NET to the standard occupational classification” (Tech. Rep. National Center for O*NET Development, Raleigh, North Carolina, 2000).

54. J. R. Spletzer, E. W. Handwerker, “Measuring the distribution of wages in the United States from 1996 through 2010 using the occupational employment survey,” in *Monthly Labor Review* (U.S. Bureau of Labor Statistics, 2014); https://doi.org/10.21916/mlr.2014.18.

55. U. S. B. of Labor Statistics, “BLS Handbook of Methods” (Bureau’s Division of BLS Publishing, Office of Publications and Special Studies, 2022).

56. E. Richards, D. Terkanian, Occupational employment projections to 2022. *Mon. Labor Rev.* **136**, 1–44 (2013).

57. S. Flood, M. King, R. Rodgers, S. Ruggles, J. R. Warren, Integrated Public Use Microdata Series, Current Population Survey: Version 8.0, IPUMS, Minneapolis (2020); https://doi.org/10.18128/D030.V8.0.

58. S. Cheng, B. Park, Flows and boundaries: A network approach to studying occupational mobility in the labor market. *Am. J. Sociol.* **126**, 577–631 (2020).

59. A. Ebenstein, A. Harrison, M. McMillan, S. Phillips, Estimating the impact of trade and offshoring on American workers using the current population surveys. *Rev. Econ. Stat.* **96**, 581–595 (2013).

60. K.-H. Lin, K. Hung, The network structure of occupations: Fragmentation, differentiation, and contagion. *Am. J. Sociol.* **127**, 1551–1601 (2022).

61. S. Flood, M. King, R. Rodgers, S. Ruggles, J. Robert Warren, D. Backman, A. Chen, G. Cooper, S. Richards, M. Schouweiler, M. Westberry, Integrated Public Use Microdata Series, Current Population Survey: Version 12.0, IPUMS, Minneapolis (2024); https://doi.org/10.18128/D030.V12.0.

62. G. Kambourov, I. Manovskii, A cautionary note on using (March) current population survey and panel study of income dynamics data to study worker mobility. *Macroeconomic Dyn.* **17**, 172–194 (2013).

63. D. McFadden, "Conditional logit analysis of qualitative choice behavior," in *Structural Analysis of Discrete Data with Econometric Applications*, P. Zarembka, Ed. (MIT Press, 1973), pp. 105–135.

64. D. McFadden, The measurement of urban travel demand. *J. Public Econ.* **3**, 303–328 (1974).

65. D. McFadden, "Modeling the choice of residential location," in Spatial Interaction Theory and Planning Models, A. Karlqvist, L. Lundqvist, F. Snickars, J. Weibull, Eds. (North Holland, 1978), pp. 75–96.

66. M. E. Ben-Akiva, S. R. Lerman, *Discrete Choice Analysis: Theory and Application to Travel Demand* (MIT Press, 1985), vol. 9.

67. J. J. Louviere, D. A. Hensher, J. D. Swait, *Stated Choice Methods: Analysis and Applications* (Cambridge Univ. Press, 2010).

68. K. E. Train, *Discrete Choice Methods with Simulation* (Cambridge Univ. Press, 2003).

69. E. E. Bruch, R. D. Mare, Methodological issues in the analysis of residential preferences, residential mobility, and neighborhood change. *Sociol. Methodol.* **42**, 103–154 (2012).

70. E. Bruch, F. Feinberg, Decision-making processes in social contexts. *Annu. Rev. Sociol.* **43**, 207–227 (2017).

71. Y. Xie, C. F. Manski, The logit model and response-based samples. *Sociol. Methods Res.* **17**, 283–302 (1989).
